# Modeling compliance specifications in linear temporal logic, event processing language and property specification patterns: a controlled experiment on understandability

**DOI:** 10.1007/s10270-019-00721-4

**Published:** 2019-02-22

**Authors:** Christoph Czepa, Amirali Amiri, Evangelos Ntentos, Uwe Zdun

**Affiliations:** Faculty of Computer Science, Research Group Software Architecture, University of Vienna, Währingerstraße 29, 1090 Wien, Austria

**Keywords:** Controlled experiment, Understandability, Linear temporal logic, Property specification patterns, Complex event processing, Event processing language

## Abstract

Mature verification and monitoring approaches, such as complex event processing and model checking, can be applied for checking compliance specifications at design time and runtime. Little is known about the understandability of the different formal and technical languages associated with these approaches. This uncertainty regarding understandability might be a major obstacle for the broad practical adoption of those techniques. This article reports a controlled experiment with 215 participants on the understandability of modeling compliance specifications in representative modeling languages, namely linear temporal logic (LTL), the complex event processing-based event processing language (EPL) and property specification patterns (PSP). The formalizations in PSP were overall more correct. That is, the pattern-based approach provides a higher level of understandability than EPL and LTL. More advanced users, however, seemingly are able to cope equally well with PSP and EPL in modeling compliance specifications.

## Introduction

Many domains are subject to a vast and ever-growing number of rules and constraints stemming from sources including laws, legislation, regulations, standards, guidelines, contracts and best practices. One example is compliance in the corporate and financial sector. The Sarbanes–Oxley Act of 2002 (SOX) [55] is a federal law that defines rules in reaction to major corporate accounting scandals in the USA (e.g., Enron and WorldCom). Basel III [4] has been established in response to weaknesses in financial regulation responsible for the financial crisis in 2007/2008. Another example of heavily regulated domains is the construction industry. Compliance rules in this domain are often related to occupational safety and health. For example, certain precautions and safe practices are required if a lead contamination is present or to be presumed in buildings built before 1978 that undergo renovation (cf. United States Environmental Protection Agency’s Lead-Based Paint Renovation, Repair and Painting Rule [83]). A third example is the healthcare sector. Processes in hospitals must comply with state-of-the-art medical knowledge and treatment procedures (e.g., Rovani et al. [71]).

From cooperations with industry partners (e.g., Tran et al. [80]), their customers and other company representatives at conferences and workshops, we were able to gain valuable insights into the current situation on how compliance rules are handled in practice. Most often, compliance documents are transformed to internal policies first. They are often described in natural language, but there is also a shift toward structured approaches like the Semantics of Business Vocabulary and Business Rules (SBVR) standard [60]. Later these internal policies become considered in business process models (e.g., BPMN [59]) or other behavioral models (e.g., UML activity diagrams), and/or they become hard-coded in a programming language. That often leads-to consistency problems and to a poor maintainability and traceability between compliance specifications, internal policies, models and the source code. This is especially the case when compliance specifications change frequently. Additionally, practitioners report that it often takes a long time until new compliance specifications are actually supported by their software. Often the compliance rule has long been obsolete before the implementation is ready (cf. [20, 48]). Consequently, the industry shows a strong interest in approaches that are applicable in practice. Such approaches should support a comprehensible, fast and accurate adoption of compliance specifications as well as their automated enactment and verification. All modeling languages that we study in this article are well suited for automated computer-aided compliance checking or monitoring. Nonetheless, companies are still often reluctant to expose their customers or employees to such approaches. In discussions with industry partners (cf. [79, 81]), uncertainty regarding how understandable these approaches are became evident. This uncertainty was stated as one of the major reasons for the reluctance in practical adoption.

### Problem statement

Most existing work on design time verification and runtime monitoring focuses on technical contributions rather than empirical contributions. From the perspective of a potential end user who has to implement compliance specifications, the understandability of an offered formal specification language appears to be a major interest. To the best of our knowledge, there are no empirical studies that investigate and compare the understandability of representative languages with respect to the formal modeling of compliance specifications. In particular, the following representative specification languages are considered in this empirical study:

- *Linear temporal logic (LTL)* was proposed in 1977 by Pnueli [65]. LTL is a popular way for defining compliance rules according to Reichert and Weber [66]. In general, LTL is a widely used specification language commonly applied in model checking (cf. Cimatti et al. [12] for NuSMV^1^, Blom et al. [9] for LTSmin^2^, Holzmann [42] for SPIN^3^) and runtime monitoring by non-deterministic finite automata (cf. De Giacomo and Vardi [23] and De Giacomo et al. [25]).
- *Event processing language (EPL)* is the query language of the open-source complex event processing engine *Esper*^4^. EPL is well suited as a representative for CEP query languages as it supports common CEP query language concepts, such as *leads-to* (*sequence*, *followed-by*) and *every* (*each*) operators, that are present in many CEP query languages and engines (e.g., Siddhi^5^ and TESLA [15]). Several existing studies on compliance monitoring make use of EPL (cf. Awad et al. [2], Holmes et al. [41] and Tran et al. [82]).
- *Property specification patterns (PSP)* are a collection of recurring temporal patterns proposed by Dwyer et al. [27, 28]. This pattern-based approach abstracts underlying technical and formal languages, most notably LTL and CTL (Computation Tree Logic; cf. Clarke et al. [13]). Numerous existing approaches are based on PSP. Among them are the *Compliance Request Language* proposed by Elgammal et al. [29] and the declarative business process approach *Declare* proposed by Pešić et al. [61].

In previous controlled experiments carried out by Czepa and Zdun [17], the understandability of already existing formal specifications in those language was studied. That experiments can be seen as the first step toward studying the understandability of those languages. To further study the understandability of these languages, it is crucial to consider the modeling itself as well.

### Research objectives

This empirical study has the research objective to investigate the understandability construct of representative languages with regard to the modeling of compliance specifications. The understandability construct focuses on the degree of correctness achieved and on the time spent on modeling compliance specifications.

The experimental goal using the goal template of the Goal Question Metric proposed by Basili et al. [5] is stated as follows:

**Analyze** LTL, PSP and EPL **for the purpose of** their evaluation **with respect to** their understandability related to modeling compliance specifications **from the viewpoint of** the novice and moderately advanced software engineer, designer or developer **in the context/environment of** the Software Engineering 2 Lab and the Advanced Software Engineering Lab courses at the Faculty of Computer Science of the University of Vienna.

Based upon the stated goal, questions concerning *understandability* were generated as shown in Table 1.

**Table 1:** Questions based upon the goal

Identifier	Question
Q1	How understandable are the tested approaches for participants at the bachelor level (attending the Software Engineering 2 Lab course)?
Q2	Are there differences in understandability between the tested approaches for participants at the bachelor level (attending the Software Engineering 2 Lab course)?
Q3	How understandable are the tested approaches for participants at the master level (attending the Advanced Software Engineering Lab course)?
Q4	Are there differences in understandability between the tested approaches for participants at the master level (attending the Advanced Software Engineering Lab course)?
Q5	How understandable are the tested approaches for participants with industrial working experience?
Q6	Are there differences in understandability between the tested approaches for participants with industrial working experience?

The *understandability* is measured by three dependent variables, namely the *syntactic correctness* and *semantic correctness* achieved in trying to formally model compliance specifications as well as the *response time*. Correctness and response time are commonly used to measure the construct understandability, for example, in empirical studies by Feigenspan et al. [31] and Hoisl et al. [40]. The study design enables a more fine-grained analysis of the correctness by differentiating between syntactic and semantic correctness as suggested by numerous existing studies, such as Ferri et al. [32], Hindawi et al. [39] and Harel and Rumpe [37].

Besides the main research goal, which focuses on understandability, this work addresses subjective aspects, namely the *perceived ease of application* and the *perceived correctness*, which are measures of self-assessment and not directly related to the understandability construct.

### Guidelines

This work follows the guidelines for reporting experiments in empirical software engineering by Jedlitschka et al. [45]. These guidelines integrate among others the “Preliminary guidelines for empirical research in software engineering” by Kitchenham et al. [50] and standard books on empirical software engineering by Wohlin et al. [86] and Juristo and Moreno [47]. The “Robust Statistical Methods for Empirical Software Engineering” article by Kitchenham et al. [49] had a strong impact on the statistical evaluation of the data in this article.

## Background

This section provides a brief introduction to the specification languages used in this study. Readers already familiar with one or more of the discussed approaches may consider skipping parts of this section. Examples of compliance specifications formalized in all three representations are available in “Appendix A.” These examples are based on the experimental tasks (cf. Sect. 3.3) of this experiment.

### Linear Temporal Logic (LTL)

Propositional logic is not expressive enough to describe temporal properties, so a logic called linear temporal logic (LTL) for reasoning over linear traces with the temporal operators ${\mathcal {G}}$ (or $\square$) for “globally” and ${\mathcal {F}}$ (or $\lozenge$) for “finally” was proposed by Pnueli [65]. Additional temporal operators are ${\mathcal {U}}$ for “until,” ${\mathcal {W}}$ for “weak until,” ${\mathcal {R}}$ for “release” and ${\mathcal {X}}$ (or $\circ$) for “next.” The meaning of these operators is described in Table 2. LTL formulas are composed of the aforementioned temporal operators, atomic propositions (the set *AP*) and the Boolean operators $\wedge$ (for “and”), $\vee$ for “or,” $\lnot$ for “not,” $\rightarrow$ for “implies” (cf. Baier and Katoen [3]). The weak-until operator $\psi ~{\mathcal {W}}~\phi$ is defined as $({\mathcal {G}}~\psi ) \vee (\psi ~{\mathcal {U}}~\phi )$.

**Table 2:** Informal meanings of LTL operators

Text notation	Symbol notation	Meaning
${\mathcal {G}}\psi$	$\square \psi$	$\psi$ must be true in every point in time
${\mathcal {F}}\psi$	$\lozenge \psi$	$\psi$ must be true at some future point in time
$\psi ~{\mathcal {U}}~\phi$	-	$\psi$ must remain true at least until the point in time when $\phi$ becomes true
$\psi ~{\mathcal {R}}~\phi$	-	$\psi$ must remain true at least until and including the point in time when $\phi$ becomes true
${\mathcal {X}}\psi$	$\circ \psi$	$\psi$ must be true at the next point in time

An LTL formula is inductively defined as follows: For every $a \in AP$, *a* is an LTL formula. If $\psi$ and $\phi$ are LTL formulas, then so are ${\mathcal {G}}\psi$ (or $\square \psi$), ${\mathcal {F}}\psi$ (or $\lozenge \psi$), $\psi ~{\mathcal {U}}~\phi$, $\psi ~{\mathcal {R}}~\phi$, ${\mathcal {X}}\psi$ (or $\circ \psi$), $\psi \wedge \phi$, $\psi \vee \phi$ and $\lnot \psi$.

The semantics of LTL over infinite traces is defined as follows: LTL formulas are interpreted as infinite words over the alphabet $2^{AP}$. The alphabet is all possible propositional interpretations of the propositional symbols in *AP*. $\pi (i)$ denotes that state of the trace $\pi$ at time instant *i*. $\pi , i \vDash \psi$ means that a trace $\pi$ at time instant *i* satisfies the LTL formula $\psi$, and is defined as follows:

- $$\pi , i \vDash a$$, for $$a \in AP$$ iff $$a \in \pi (i)$$.
- $$\pi , i \vDash \lnot \psi $$ iff $$\pi , i \nvDash \psi $$.
- $$\pi , i \vDash \psi \wedge \phi $$ iff $$\pi , i \vDash \psi $$ and $$\pi , i \vDash \phi $$.
- $$\pi , i \vDash \psi \vee \phi $$ iff $$\pi , i \vDash \psi $$ or $$\pi , i \vDash \phi $$.
- $$\pi , i \vDash {\mathcal {X}} \psi $$ iff $$\pi , i+1 \vDash \psi $$.
- $\pi , i \vDash {\mathcal {F}} \psi$ iff $\exists j \ge i$, such that $\pi , j \vDash \psi$.
- $\pi , i \vDash {\mathcal {G}} \psi$ iff $\forall j \ge i$, such that $\pi , j \vDash \psi$.
- $\pi , i \vDash \psi ~{\mathcal {U}}~\phi$ iff $\exists j \ge i$, such that $\pi , j \vDash \phi$ and $\forall k, i \le k < j$, we have $\pi , k \vDash \psi$.
- $\pi , i \vDash \psi ~{\mathcal {R}}~\phi$ iff $\forall j \ge i$, iff $\pi , j \nvDash \phi$, then $\exists k, i \le k < j$, such that $\pi , k \vDash \psi$.

For the definition of the semantics of LTL over finite traces, we refer the interested reader to the work of De Giacomo and Vardi [23] and De Giacomo et al. [25].

In model checking, LTL formulas commonly have two possible truth value states, namely true and false. In case of monitoring a compliance specification in a running system, it might be the case, that it is not only of interest if it is satisfied or violated but also whether further state changes are possible that could resolve or cause a violation of it. That is, the runtime state of a specification is either *temporary* or *permanent*. Consequently, an LTL specification at runtime is either temporarily satisfied, temporarily violated, permanently satisfied or permanently violated (cf. Bauer et al. [6, 7]). Several existing studies make use of the concept of four LTL truth value states (cf. Pešić et al. [62], De Giacomo et al. [24] and Maggi et al. [54]).

### Event Processing Language (EPL)

In this section, the event processing language (EPL) [30] is discussed and how it can be applied for runtime monitoring of compliance specifications. An EPL-based specification consists of an initial truth value, which is either assigned to temporarily satisfied or temporarilyviolated, and one or more query–listener pairs. A query–listener pair causes a truth value change in the specification as soon as a matching event pattern is observed in the event stream. Consequently, an EPL-based compliance specification always consists of EPL queries that are composed of EPL operators and listeners that cause truth value changes to temporarily satisfied, temporarily violated, permanently satisfied, permanently violated, as already discussed for LTL in Sect. 2.1. The truth value state of the specification is updated by a positive match of the related expression in the event stream. Based on the notation suggested by Czepa et al. [18, 19], the short notation $\texttt {<EPL query> ==> <truth} \mathtt{value{>}}$ is used for an EPL query–listener pair responsible for changing the truth value of a compliance rule. Obviously, further truth value changes are not possible once a permanent state, namely either permanently violated or permanently satisfied, has been reached. According to the EPL reference [30], the semantics is given as shown in Table 3.

**Table 3:** Semantics of EPL operators

Operator name	Representation	Semantics
and	$e_1$ and $e_2$	Logical conjunction that is matched once both $e_1$ and $e_2$ in any order have occurred
or	$e_1$ or $e_2$	Logical disjunction that is matched once either $e_1$ or $e_2$ has occurred
not	not *e*	Logical negation that is matched if the expression *e* is not matched
every	every *e*	Not just observe the first occurrence of the expression *e* in the event stream but also each subsequent one
leads-to	$e_1$ -> $e_2$	The first $e_1$ must be observed and only then is $e_2$ matched. Intuitively, the whole expression is matched once $e_1$ is followed by $e_2$ at the occurrence of $e_2$
until	$e_1$ until $e_2$	Matches the expression $e_1$ until $e_2$ occurs. In practice, this operator is commonly used in the expression not$e_1$until$e_2$ that demands the absence of $e_1$ before the occurrence of $e_2$

### Property specification patterns (PSP)

**Fig. 1:**
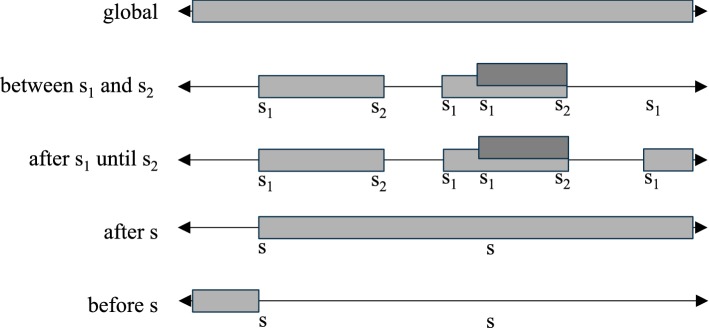
Available scopes for property specification patterns (shaded areas indicate the extent over which the pattern must hold)

Dwyer et al. proposed the property specification patterns (PSP) [27, 28], a collection of recurring specification patterns. For each pattern, there exist transformation rules to underlying formal representations , including LTL and CTL^6^. The patterns are categorized into *Occurrence Patterns* and *Order Patterns* as shown in Tables 4 and 5, respectively. Figure 1 shows the area of effect of available scopes, whereas Table 6 discusses their meaning.

**Table 4:** Intents of occurrence patterns

Pattern name	Representation	Intent
Absence	*a* never occurs	To describe a portion of a system’s execution that is free of certain events or states
Universality	*a* always occurs	To describe a portion of a system’s execution which contains only states that have a desired property
Existence	*a* occurs	To describe a portion of a system’s execution that contains an instance of certain events or states
Bounded existence	*a* occurs at most *n* times	To describe a portion of a system’s execution that contains at most a specified number of instances of a designated state transition or event

**Table 5:** Intents of order patterns

Pattern name	Representation	Intent
Precedence	*a* precedes *b*	To describe a relationship between a pair of events/states where the occurrence of the first is a necessary precondition for an occurrence of the second
Response	*a* leads-to *b*	To describe a cause–effect relationship between a pair of events/states. An occurrence of the first, the cause, must be followed by an occurrence of the second, the effect
2 Cause–1 Effect Precedence Chain	(*a*, *b*) precedes*c*	To describe a relationship between an event/state sequence (*a*, *b*) and an event/state *c* in which the occurrence of *c* within the scope must be preceded by a sequence of events/states (*a*, *b*) within the same scope
1 Cause–2 Effect Precedence Chain	*a*precedes (*b*, *c*)	To describe a relationship between an event/state *a* and a sequence of events/states (*b*, *c*) in which the occurrence of *b* followed by *c* within the scope must be preceded by an occurrence of *a* within the same scope
2 Stimulus–1 Response Chain	(*a*, *b*) leads-to*c*	To describe a relationship between a stimulus sequence (*a*, *b*) and a response event *c* in which the occurrence of the stimulus events must be followed by an occurrence of the response event within the scope
1 Stimulus–2 Response Chain	*a*leads-to (*b*, *c*)	To describe a relationship between a stimulus event *a* and a sequence of two response events (*b*, *c*) in which the occurrence of the stimulus event must be followed by an occurrence of the sequence of response events within the scope

The available runtime states of PSP specifications are no different from those of LTL and EPL specifications (cf. Sects. 2.1 and 2.2), namely temporarily satisfied, temporarily violated, permanentlysatisfied and permanently violated.

## Experiment planning

This section describes the outcome of the experiment planning phase, and it provides all information that is required for a replication of the study.

### Goals

The primary goal of the experiment is measuring the construct *understandability* of representative languages that are suitable for modeling compliance specifications. This construct is defined by the *syntactic correctness*, *semantic correctness* and *response time* of the answers given by the participants.

This study differentiates between syntactic and semantic correctness as it enables a more fine-grained analysis. This is in line with Chomsky [11], who stressed that the study of syntax must be independent from the study of semantics. Numerous existing studies differentiate between syntactic and semantic correctness (cf. Ferri et al. [32], Hindawi et al. [39] and Harel and Rumpe [37]). On the other hand, an LTL formula can be syntactically totally correct without catching the desired meaning. For example, the specification “activity 2 must not happen unless activity 1 has already happened” is not covered at all in a semantic way by the syntactically correct formula “${\mathcal {F}}~activity_1~\wedge ~{\mathcal {F}}~activity_2$.” In contrast, the formula “$\lnot ~activity_2~{\mathcal {U}}~activity_1$” is both syntactically and semantically correct.

**Table 6:** Meaning of scopes

Scope name	Representation	Meaning
global	This scope is implicitly assumed when no other scope is defined	Defines that a pattern must hold during the entire execution of a system
before	before *s* [ *p* ]	*p* must hold before the first occurrence of *s*
after	after *s* [ *p* ]	*p* must hold after the first occurrence of *s*
between	between $s_1$ and $s_2$ [ *p* ]	*p* must hold between every $s_1$ (i.e., starting the scope) that is followed by $s_2$ (i.e., closing the scope)
after-until	after $s_1$ until $s_2$ [ *p* ]	*p* must hold after every $s_1$ (i.e., starting the scope) by no later than $s_2$ (i.e., closing the scope)

In addition to the understandability construct, the experiment aims at studying the *perceived ease of application* of the languages and the *perceived correctness* of the formalized compliance specifications.

### Experimental units

All 215 participants of the experiment are students who enrolled in the courses “Software Engineering Lab (SE2)” and “Advanced Software Engineering Lab (ASE)” at the Faculty of Computer Science, University of Vienna, Austria. Two kinds of participants can be differentiated:

- 149 participants of the bachelor-level course SE2 are used as proxies for *novice* software engineers, designers or developers.
- 66 participants of the master-level course ASE are used as proxies for *moderately advanced* software engineers, designers or developers.

Using students as proxies for non-expert users is not an issue according to Kitchenham et al. [50]. Other studies even suggest that students can be used as proxies for experts under certain circumstances (cf. Höst et al. [43], Runeson [72], Svahnberg et al. [78] and Salman et al. [73]). As an incentive for participation and proper preparation, up to 10 bonus points ($10 \%$ of total course points) were awarded based on the participant’s performance in the experiment. All participants were randomly allocated to experiment groups.

### Experimental material and tasks

**Fig. 2:**
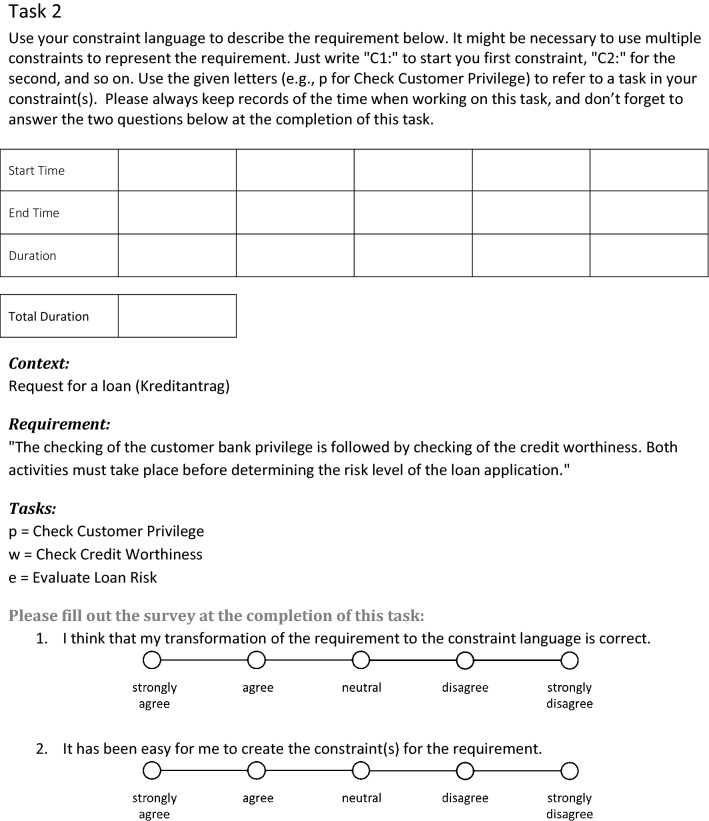
Sample task as presented to the participants

In total, the experiment comprised five distinct tasks stemming from three different domains, as shown in Table 7. Tasks 1 and 2 are related to compliance in the context of lending, Task 3 focuses on compliance regarding hospital processes, and Tasks 4 and 5 are based on compliance specifications in the construction industry. Each task was presented to the participants by stating first the context, then the specification and last the available elements that are to be used during formal modeling of the specification. For an example, how experimental tasks were presented to the participants, see Fig.2. The full experimental material is available online (cf. Czepa et al. [22]). For sample solutions of all experimental tasks, see “Appendix A.” It is important to note that these sample solutions show just one way to model the compliance specifications. In the grading process, each proposed solution was carefully assessed under constant consideration that the sample solution might not be the only way to correctly formalize the specification.

**Table 7:** Experimental tasks

Task No.	Context/Source	Compliance specification in natural language	Available elements for modeling
1	Request for a loan (cf. Elgammal et al. [29])	The branch office manager has to evaluate the loan risk before signing the contract officially. No one else is allowed to evaluate the loan risk and to sign the contract	Tasks
			Evaluate loan risk
			Officially sign contract
			Roles
			Branch office manager
2	Request for a loan (cf. Elgammal et al. [29])	The checking of the customer bank privilege is followed by checking of the credit worthiness. Both activities must take place before determining the risk level of the loan application	Tasks
			Check customer privilege
			Check credit worthiness
			Evaluate loan risk
3	Medical treatment and surgery of malignant gastric diseases (cf. Rovani et al. [71])	The preoperative screening is performed before any surgical treatment in order to assess whether the patient’s conditions are good enough for the surgery to be performed and to estimate potential risks. As far as the surgical technique is concerned, the gastric resection for malignant diseases can be performed by using either a laparoscopic surgery or a traditional open approach, but not both. Furthermore, in both cases a nursing period is needed to monitor the patient after the operation	Tasks
			Preoperative screening
			Laparoscopic gastrectomy
			Open gastrectomy
			Nursing
4	Renovation work and lead-based paint (cf. United States Environmental Protection Agency [83])	Once a lead contamination has been identified, a certified renovator must be present all time while any cleaning activity is performed until the end of the renovation work	Tasks
			Renovation
			Cleaning
			Presence of certified renovator
			Events
			Lead contamination identified
5	Renovation work and lead-based paint (cf. United States Environmental Protection Agency [83])	Contractors, property managers and others who perform renovations for compensation in residential houses, apartments and child-occupied facilities built before 1978 are required to distribute a lead pamphlet before starting renovation work	Tasks
			Renovation
			Distribute lead pamphlet
			Classify building
			Enter building date
			Data
			Year of construction
			Type of building

### Hypotheses, parameters and variables

PSP abstracts underlying formal representations, such as LTL formulas, by high-level patterns with the intention to facilitate reuse and to enable ease of use. That is, the pattern representations are assumed to provide a better understandability than their underlying LTL formulas. EPL-based constraints are composed of an initial truth value and one or more query–listener pairs that change the truth value state. In contrast to LTL where meaning is encoded in a formula, different concerns, namely defining the initial truth value and change criteria for the truth value, are separated from each other in EPL-based constraints. This separation of concerns is assumed to facilitate the understandability of EPL-based constraints as opposed to LTL formulas where this separation is not present.

Consequently, we hypothesized that PSP, as a highly abstract pattern language, is easier to understand than LTL and EPL and that EPL, due to separation of concerns, is easier to understand than LTL. Consequently, the following hypotheses for the controlled experiment were formulated:

- $H_{0,1}$: There is no difference in terms of *understandability* between PSP and LTL.
- $H_{A,1}$: PSP has a higher level of *understandability* than LTL.
- $H_{0,2}$: There is no difference in terms of *understandability* between PSP and EPL.
- $H_{A,2}$: PSP has a higher level of *understandability* than EPL.
- $H_{0,3}$: There is no difference in terms of *understandability* between EPL and LTL.
- $H_{A,3}$: EPL has a higher level of *understandability* than LTL.

The construct *understandability* is measured by three interval-scaled dependent variables, namely:

- the *syntactic correctness* achieved in trying to formally model the compliance specifications,
- the *semantic correctness* achieved in trying to formally model the compliance specifications,
- the *response time*, which is the time it took to complete the experimental tasks.

In addition, there are hypotheses that are concerned with the participants’ opinion on the languages under investigation, namely:

- $H_{0,4}$: There is no difference in terms of *perceived correctness* between PSP and LTL.
- $H_{A,4}$: PSP has a higher level of *perceived correctness* than LTL.
- $H_{0,5}$: There is no difference in terms of *perceived correctness* between PSP and EPL.
- $H_{A,5}$: PSP has a higher level of *perceived correctness* than EPL.
- $H_{0,6}$: There is no difference in terms of *perceived correctness* between EPL and LTL.
- $H_{A,6}$: EPL has a higher level of *perceived correctness* than LTL.
- $H_{0,7}$: There is no difference in terms of *perceived ease of application* between PSP and LTL.
- $H_{A,7}$: PSP has a higher level of *perceived ease of application* than LTL.
- $H_{0,8}$: There is no difference in terms of *perceived ease of application* between PSP and EPL.
- $H_{A,8}$: PSP has a higher level of *perceived ease of application* than EPL.
- $H_{0,9}$: There is no difference in terms of *perceived ease of application* between EPL and LTL.
- $H_{A,9}$: EPL has a higher level of *perceived ease of application* than LTL.

The dependent variables associated with these hypotheses are ordinal scaled since the data were gathered by agree–disagree scales. In accordance with the results of a study by Revilla et al. [68], each scale had five categories.

### Experiment design and execution

According to Wohlin et al. [86], “it is important to try to use a simple design and try to make the best possible use of the available subjects.” For that reason, a completely randomized experiment design with one alternative per experimental unit was used. That is, each participant is randomly assigned to exactly one experiment group. This assignment took place fully automated in an unbiased manner.

Preparation documents were distributed to the participants one week before the experiment run. In these documents, the basics of the approaches are discussed, and the participants were encouraged to prepare for the experiment by applying the assigned behavioral constraint representation before the experiment session. To avoid bias, all three preparation documents are similar in length and depth. The approaches were presented in an approachable manner to the participants as suggested by numerous existing research on teaching undergraduate students in theoretical computer science, formal methods and logic (cf. Habiballa and Kmeť [34], Knobelsdorf and Frede [51], Carew et al. [10] and Spichkova [77]). The used training material is available online (cf. Czepa et al. [22]).

### Procedure

**Fig. 3:**
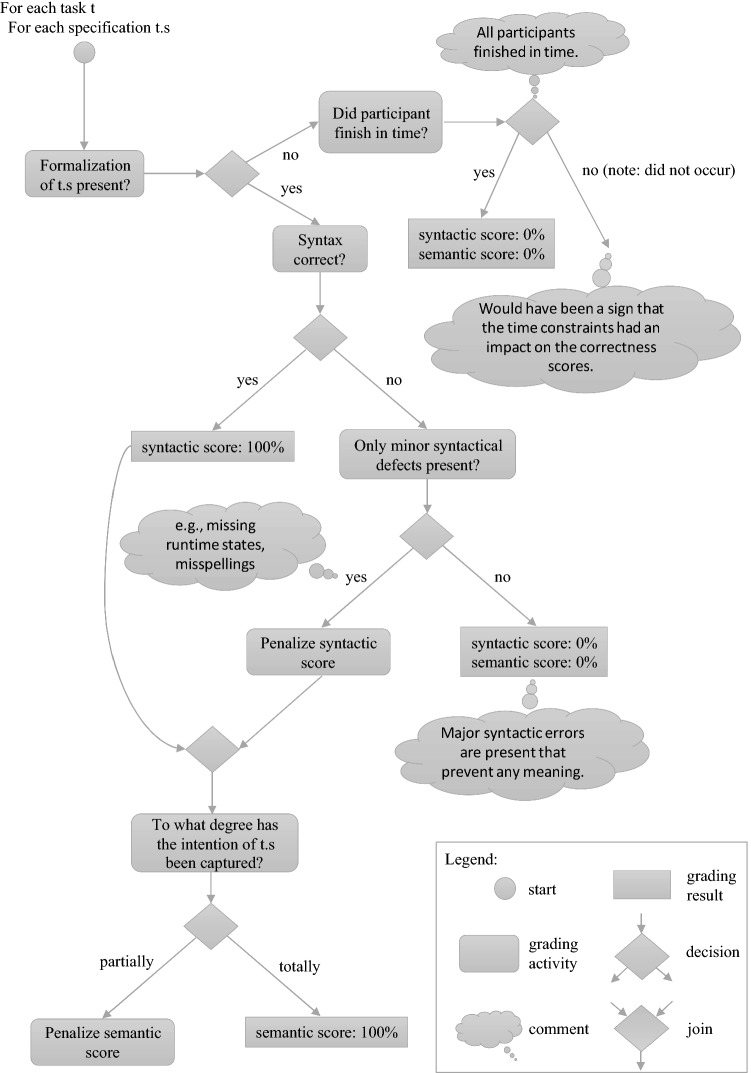
Individual grading procedure

**Fig. 4:**
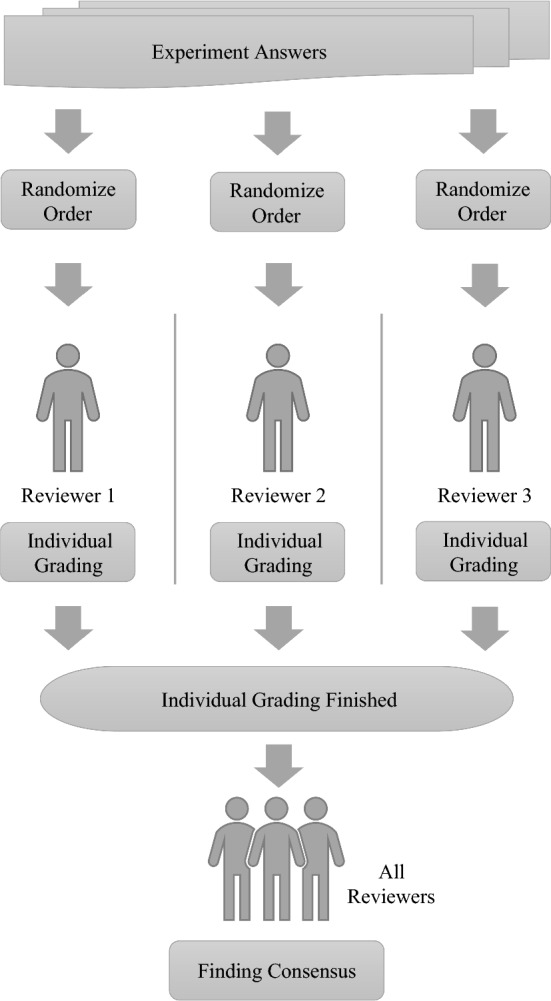
Overall grading procedure

To ensure a smooth procedure and to avoid unnecessary stress, the preparation document informed the participants about the procedure on the experiment day as detailed as possible. Seating arrangements were made to limit chances for misbehavior, and the participants were instructed how to find a suitable seat. The participants were allowed to use printouts of the preparation material and notes at their own discretion. After a brief discussion of the contents and structure of the experiment document by the experimenters, the participants started trying to solve the experimental tasks. The duration of the experiment was limited to 90 min. Due to organizational reasons, the experiment was done on paper, and time record keeping was the responsibility of each participant (please see Sect. 5.2 for a discussion of this potential threat to validity). After experiment execution, the answers given were evaluated. For that purpose, a method proposed by Lytra et al. [53] was applied, which comprises the independent evaluation of the answers by three experts, and a discussion of large differences in grading until a consensus is achieved. The attempted formalization in each experiment tasks was graded independently by the first, second and third author, who are experts in the investigated languages. To mitigate the risk of grading bias, the participant’s given answers were graded in random order by each of the experts, and, in case of large differences in grading, a discussion took place until a consensus was achieved. Figures 3 and 4 depict the grading process schematically from the individual and overall perspective, respectively. This evaluation of more than a thousand distinct answers comprising approximately 17,000 constraints took about half a year besides the authors’ normal responsibilities such as teaching and other research. All other given answers, which are related to previous knowledge, time records and agree–disagree scale responses, were digitized and double-checked subsequently.

## Analysis

This section is concerned with the treatment and statistics of the data.

### Data set preparation

To preserve the integrity of the acquired data, it was necessary to drop potentially unreliable items. In total, the data of eight participants were not considered in the statistical evaluations. Table 8 summarizes all dropped participants including the reasons for non-consideration.

### Descriptive statistics

In this section, the acquired data (cf. Czepa et al. [22]) are analyzed by the help of descriptive statistics.

Table 9 shows the number of observations, central tendency and dispersion of the dependent variables *syntactic correctness*, *semantic correctness* and *response time* per group. In the bachelor-level course *Software Engineering 2*, the sample size is relatively large and evenly distributed (9 : 47 : 49). In the master-level course *Advanced Software Engineering*, there are less than half as many observations. Unfortunately, the number of participants of the group with the smallest number of observations, namely PSP, was further diminished by the exclusion of three participants (cf. Sect. 4.1). In consequence, the distribution in the ASE course is 21 : 17 : 24. The median and mean correctness values of the LTL groups in both SE2 and ASE are smaller than those of the other two groups. In SE2, the mean syntactic correctness of the LTL group is 56.52, thus about $5 \%$ less than in the EPL group ($61.82 \%$) and about $12 \%$ less than in the PSP group ($68.64 \%$), and the mean semantic correctness of the LTL group is at $28.49 \%$, so about $10 \%$ below the EPL group ($38.20 \%$) and $22 \%$ below the PSP group ($50.19 \%$). In ASE, the mean syntactic correctness of the LTL group is $57.01 \%$, thus about $8 \%$ less than in the PSP group ($65.13 \%$) and about $15 \%$ less than in the EPL group ($71.91 \%$). While the PSP group overall achieved a higher syntactic and semantic correctness than the LTL group in SE2, this ranking is reversed in the ASE course where EPL participants overall achieved a higher syntactic and semantic correctness than their colleagues of the PSP group. The mean syntactic correctness achieved by the PSP group ($65.13 \%$) is about $7 \%$ higher than in the EPL group ($71.91 \%$) in SE2, whereas the EPL group achieved an about $7 \%$ higher mean syntactic correctness ($71.91 \%$) than the PSP group ($65.13 \%$) in ASE. In SE2, the mean semantic correctness of the PSP group ($50.19 \%$) is about $12 \%$ higher than in the EPL group ($38.20 \%$). In ASE, the mean semantic correctness is about $3 \%$ higher in the EPL group ($49.71 \%$) than in the PSP group ($46.93 \%$). The mean and median response times are overall faster in the SE2 course than in the ASE course. In SE2, the mean response time of the LTL group (43.49 min) is slightly faster than in EPL (44.87 min) and a few minutes faster than in the PSP group (48.68 min). In ASE, the mean response time of the LTL group (52.32 min) is 3–4 min faster than in the PSP group (55.99 min) and 6–7 min faster than in the EPL group (58.82 min).

*Skew* is a measure of the shape of a distribution. A positive skew value indicates a right-tailed distribution (e.g., more cases of low correctness than high correctness), a negative skew value indicates a left-tailed distribution (e.g., more cases of high correctness than low correctness), and a skew value close to zero indicates a symmetric distribution. Differences in skew are, for example, present

- between the semantic correctness distributions of LTL (0.75 indicating that the mass of the distribution is concentrated at lower levels of correctness) and PSP ($-\,0.08$ indicating a rather symmetric distribution) in SE2,
- between the syntactic correctness distributions of LTL ($-0.15$ indicating a curve that is slightly leaned to the right) and EPL ($-0.9$ indicating a distribution with only few measurements in lower correctness ranges) in ASE,
- between the semantic correctness distributions of LTL (0.6 indicating higher densities in lower correctness ranges) and EPL ($-0.37$ indicating higher densities in higher correctness ranges) in ASE, and
- between the response time distributions of LTL (0.42 indicating a left-leaning curve) and PSP ($-0.61$ indicating a right-leaning curve) in ASE.

**Table 8:** Summary of dropped participants

Group	Course	Reason
PSP	SE2	The participant gave up after the first task
PSP	SE2	The participant did not apply PSP, but used a language/formalism that was not part of the study
LTL	SE2	The participant was assigned to LTL, but gave answers in PSP
LTL	SE2	The participant gave positive perceived difficulty and correctness ratings for unsolved tasks
PSP	ASE	The participant did not apply PSP, but wrote basic Boolean formulas
PSP	ASE	The participant came unprepared
PSP	ASE	The participant did not apply PSP, but drew UML activity diagrams
LTL	ASE	The participant gave up after the first task

**Table 9:** Number of observations, central tendency and dispersion of the dependent variables semantic/syntactic correctness and response time per group and course

	LTL	PSP	EPL
Total number of observations	51	49	49
Number of considered observations	49	47	49
*Software Engineering 2 (SE2) (bachelor-level course)*			
Syntactic correctness			
Arithmetic mean (%)	56.52	68.64	61.82
Standard deviation (SD) (%)	16.40	16.99	16.85
Median (%)	57.84	72.55	61.76
Median absolute deviation (MAD) (%)	19.19	13.37	18.61
Minimum (%)	9.02	24.51	21.18
Maximum (%)	96.27	98.82	89.22
Skew	$-0.3$	$-0.55$	$-0.53$
Kurtosis	0.01	$-0.09$	$-0.4$
Semantic correctness			
Arithmetic mean (%)	28.49	50.19	38.20
Standard deviation (SD) (%)	14.48	15.74	14.73
Median (%)	27.06	49.61	36.08
Median absolute deviation (MAD) (%)	13.66	15.12	13.66
Minimum (%)	2.75	18.04	10
Maximum (%)	68.43	80.59	72.55
Skew	0.75	$-0.08$	0.27
Kurtosis	0.24	$-0.68$	$-0.56$
Response time			
Arithmetic mean (min)	43.49	48.68	44.87
Standard deviation (SD) (min)	13.10	14.39	14.07
Median (min)	40.50	45.67	47.22
Median absolute deviation (MAD) (min)	11.98	17.49	13.66
Minimum (min)	15.07	27.00	14.58
Maximum (min)	75.40	79.93	75.00
Skew	0.33	0.38	0.14
Kurtosis	$-0.35$	$-0.93$	$-0.41$
Total number of observations	22	20	24
Number of considered observations	21	17	24
*Advanced Software Engineering (ASE) (master-level course)*			
Syntactic correctness			
Arithmetic mean (%)	57.01	65.13	71.91
Standard deviation (SD) (%)	15.62	21.02	13.78
Median (%)	56.67	67.84	72.06
Median absolute deviation (MAD) (%)	18.90	26.74	10.47
Minimum (%)	29.61	21.76	31.76
Maximum (%)	81.96	89.41	94.71
Skew	$-0.15$	$-0.5$	$-0.9$
Kurtosis	1.22	$-1.02$	1.05
Semantic correctness			
Arithmetic mean (%)	30.85	46.93	49.71
Standard deviation (SD) (%)	12.96	17.14	13.46
Median (%)	29.61	47.84	51.57
Median absolute deviation (MAD) (%)	14.54	19.19	12.06
Minimum (%)	12.75	17.65	19.41
Maximum (%)	63.14	75.69	76.86
Skew	0.6	0.06	$-0.37$
Kurtosis	$-0.41$	$-1.12$	$-0.45$
Response time			
Arithmetic mean (min)	52.32	55.99	58.82
Standard deviation (SD) (min)	15.36	13.64	14.15
Median (min)	49.00	62.00	58.00
Median absolute deviation (MAD) (min)	16.88	11.64	15.64
Minimum (min)	28.00	29.50	37.17
Maximum (min)	84.00	73.08	81.78
Skew	0.42	$-0.61$	0.15
Kurtosis	$-0.94$	$-1.09$	$-1.19$

*Kurtosis* is another measure for the shape of a distribution which focuses on the general tailedness. Positive kurtosis values indicate skinny tails with a steep distribution, whereas negative kurtosis values indicate fat tails. The most severe difference in kurtosis is present between the syntactic correctness distributions of the LTL group (1.22) and PSP group ($-1.02$).

So far, the dependent variables were analyzed on the basis of separating between course groups, which reflects the participants academic level of progression. Next, the dependent variables are investigated focusing on participants with industrial working experience. Table 10 summarizes the descriptive statistics of the dependent variables when focusing on participants with industrial working experience of one year and above. Based on the demographic data collected (cf. “Appendix D”), we consider this subset of participants to be close to the population of industrial practitioners with basic to modest experience. The mean syntactic correctness in the LTL group ($58.65 \%$) is about $8 \%$ lower than in the PSP ($66.79 \%$) and EPL ($66.01 \%$) groups. The PSP group achieved the highest degree of semantic correctness ($48.58 \%$), closely followed by the EPL group ($44.46 \%$). The LTL group achieved $30.51 \%$ semantic correctness, which is noticeable lower than in the two other groups. Present differences in skew and kurtosis are indications of differences in central location and distribution shape.

**Table 10:** Number of observations, central tendency and dispersion of the dependent variables semantic/syntactic correctness and response time per group of participants with working experience $\ge 1$ year

	LTL	PSP	EPL
Number of observations	20	17	22
Syntactic correctness			
Arithmetic mean (%)	58.65	66.79	66.01
Standard deviation (SD) (%)	14.68	17.76	14.82
Median (%)	58.82	67.84	70.20
Median absolute deviation (MAD) (%)	16.42	13.08	12.50
Minimum (%)	31.18	21.76	26.67
Maximum (%)	81.96	89.41	89.22
Skew	$-$ 0.33	$-$ 0.89	$-$ 0.87
Kurtosis	$-$ 1.03	0.24	0.32
Semantic correctness			
Arithmetic mean (%)	30.51	48.58	44.46
Standard deviation (SD) (%)	16.04	16.93	15.20
Median (%)	28.73	49.22	45.78
Median absolute deviation (MAD) (%)	16.86	20.93	18.46
Mnimum (%)	8.24	17.65	15.69
Maximum (%)	63.33	75.69	72.55
Skew	0.55	0.2	$-$ 0.1
Kurtosis	$-$ 0.72	$-$ 1.27	$-$ 1.07
Response time			
Arithmetic mean (min)	49.31	49.19	48.64
Standard deviation (SD) (min)	16.81	13.34	14.03
Median (min)	47.94	48.85	48.13
Median absolute deviation (MAD) (min)	15.80	20.36	15.52
Minimum (min)	15.07	29.50	24.07
Maximum (min)	84.00	66.00	76.08
Skew	0.29	0.21	0.22
Kurtosis	$-$ 0.42	$-$ 1.56	$-$ 0.87

**Fig. 5:**
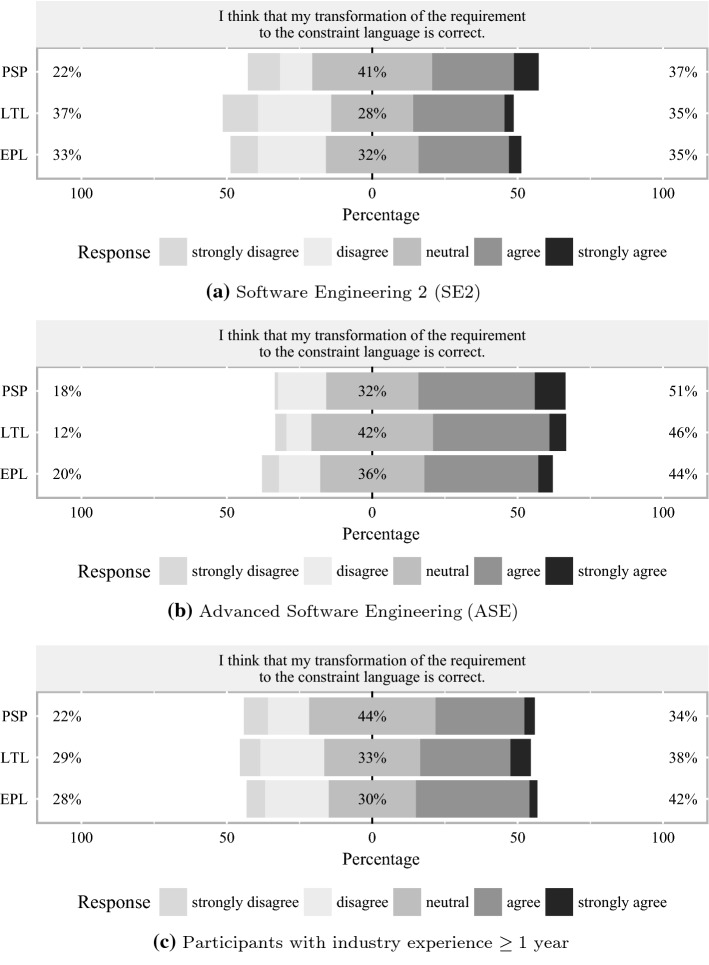
Participants’ perceived correctness

For additional descriptive statistics of the dependent variables *syntactic correctness, semantic correctness and response time*, we refer the interested reader to “Appendix B.”

With regard to the stacked bar chart (cf. Bryer and Speerschneider [44]) in Fig.5a showing the *perceived correctness* in SE2, the share of strongly agree responses to the statement “I think that my transformation of the requirement to the constraint language is correct” is $2 \%$ higher in PSP ($37 \%$) than in the other two groups, and the share of (strongly) disagree answers is $22 \%$ in PSP while it is higher in LTL ($37 \%$) and EPL ($33 \%$). With $41 \%$ the share of neutral answers is largest in PSP. In ASE (cf. Fig. 5b), the participants appear to be overall slightly more confident regarding the correctness of their formalizations. The largest share of (strongly) agree responses is again present in the PSP group ($51 \%$), followed by LTL ($46 \%$) and EPL ($44 \%$). According to the stacked bar charts in Fig. 5, the perceived correctness of PSP appears to be slightly higher than in the other experiment groups in SE2, while EPL has a slightly lower perceived correctness than the other languages in ASE. According to Fig. 5c, a large share ($44 \%$) of participants with industry experience in the PSP is undecided whether the given answer is correct. The percentage of neutral answers of participants with industry experience is lowest in the EPL group ($30 \%$) and only slightly higher in the LTL group. The largest share of (strongly) agree responses of participants with industry experience is present in the EPL group ($42 \%$), followed by LTL ($38 \%$) and PSP ($34 \%$).

**Fig. 6:**
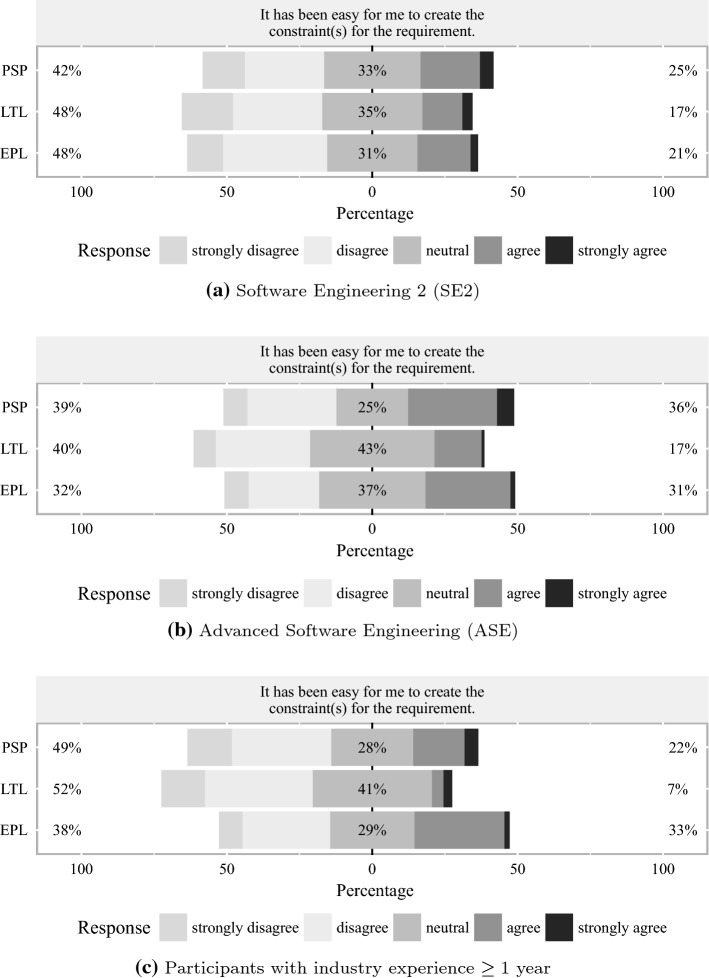
Participants’ perceived ease of application

Figure 6 contains stacked bar charts of the participants’ *perceived ease of application* of the tested languages. Interestingly, there appears to be a strong similarity between the *perceived correctness* and *perceived ease of application* responses in SE2 regarding the ranking of the approaches (cf. Figs. 6a, 5a). PSP with $25 \%$ (strongly) agreeing and $42 \%$ (strongly) disagreeing appears to be slightly easier to apply than EPL with $21 \%$ (strongly) agreeing and $48 \%$ (strongly) disagreeing, and LTL with $17 \%$ (strongly) agreeing and $48 \%$ (strongly) disagreeing is perceived slightly more difficult to apply than EPL. In ASE (cf. Fig. 6b), the application of PSP is perceived to be even easier than in SE2. Interestingly, EPL is perceived to be similarly easy as PSP with regard to application. Like in SE2, LTL is ranked last in *perceived ease of application*. Figure 6c focuses on industry participants and reveals striking differences between the groups. The perceived ease of application is highest rated in the EPL group with $33 \%$ (strongly) agreeing and $38 \%$ (strongly) disagreeing, which means that there is still a shift toward a negative rating. The strongest shift toward low ease of application is present in the LTL group with only $7 \%$ (strongly) agreeing and $52 \%$ (strongly) disagreeing. In between are the results of the PSP group with $22 \%$ (strongly) agreeing and $49 \%$ (strongly) disagreeing.

### Statistical inference

Before applying any statistical test, its model assumption must be tested and met. For a discussion whether or not the normality assumption is violated by the acquired data, see “Appendix C.” Since there is uncertainty regarding normality, a core assumption of parametric testing, nonparametric testing is the preferable approach.

**Table 11:** Cliff’s *d* of syntactic/semantic correctness and response time in SE2, one-tailed with confidence intervals calculated for $\alpha = 0.05$ (cf. Cliff [14] and Rogmann [70]), adjusted p-values (cf. Benjamini and Hochberg [8]) [level of significance: * for $\alpha = 0.05$, ** for $\alpha = 0.01$, *** for $\alpha = 0.001$] and effect size magnitudes (cf. Kitchenham et al. [49])

	PSP/LTL	PSP/EPL	EPL/LTL
Syntactic correctness			
$p_1 = P(X>Y)$	0.7059	0.6071	0.6028
$p_2 = P(X=Y)$	0.0038	0.0014	0.0046
$p_3 = P(X<Y)$	0.2904	0.3916	0.3926
*d*	$-0.4155$	$-0.2155$	$-0.2103$
$s_d$	0.1054	0.1166	0.1148
*z*	$-3.9412$	$-1.8477$	$-1.8308$
CI low	$-0.5733$	$-0.3976$	$-0.3899$
CI high	$-0.2281$	$-0.0171$	$-0.0152$
*p*	$7.7 \times 10^{-5}$	0.0339	0.0351
FDR adjusted *p*	0.0004	0.0658	0.0658
Level of significance	***	–	–
Effect size magnitude	Medium	–	–
Semantic correctness			
$p_1 = P(X>Y)$	0.8448	0.7153	0.6913
$p_2 = P(X=Y)$	0.1356	0.0032	0.0058
$p_3 = P(X<Y)$	0.1535	0.2816	0.3029
*d*	$-0.6913$	$-0.4337$	$-0.3884$
$s_d$	0.0794	0.1057	0.1066
*z*	$-8.7104$	$-4.1028$	$-3.6445$
CI low	$-0.8006$	$-0.5909$	$-0.549$
CI high	$-0.5374$	$-0.2447$	$-0.2002$
*p*	$4.4 \times 10^{-14}$	$4.4 \times 10^{-5}$	0.0002
FDR adjusted *p*	$6.5 \times 10^{-13}$	0.0003	0.0008
Level of significance	***	***	***
Effect size magnitude	Large	Large	Medium
Response time			
$p_1 = P(X>Y)$	0.5928	0.5632	0.5298
$p_2 = P(X=Y)$	0.0017	0.0023	0.0029
$p_3 = P(X<Y)$	0.4055	0.4346	0.4673
*d*	0.1873	$-0.1286$	$-0.0626$
$s_d$	0.1153	0.119	0.1185
*z*	1.625	$-1.0808$	$-0.5281$
CI low	$-0.3685$	$-0.3176$	$-0.2533$
CI high	0.0076	0.0702	0.1329
*p*	0.0537	0.1413	0.2993
FDR adjusted *p*	0.0895	0.1766	0.2993
Level of significance	–	–	–
Effect size magnitude	–	–	–

Standard nonparametric tests like Kruskal–Wallis cannot be applied if distribution shapes differ apart from their central location (cf. descriptive statistics in “Appendix B”), so *Cliff’s delta* (cf. Cliff [14] and Rogmann [70]), a robust nonparametric test, is applied. Table 11 summarizes the test results for the bachelor-level course SE2. To take multiple testing into account, the p-values are adjusted based on the method proposed by Benjamini and Hochberg [8]. There is a highly significant result with a medium effect size magnitude, indicating that PSP provides a higher syntactic correctness than LTL. After p-value adjustments, no such result is present in the remaining syntactic correctness tests. All semantic correctness test results are highly significant with medium- to large-sized effects. There is no significant difference between the response times. Consequently, $H_{0,1}$ is rejected on the basis of syntactic and semantic correctness whereas $H_{0,2}$ and $H_{0,3}$ can only be rejected based on semantic correctness.

**Table 12:** Cliff’s *d* of syntactic/semantic correctness and response time in ASE, one-tailed with confidence intervals calculated for $\alpha = 0.05$ (cf. Cliff [14] and Rogmann [70]), adjusted p-values (cf. Benjamini and Hochberg [8]) [level of significance: * for $\alpha = 0.05$, ** for $\alpha = 0.01$, *** for $\alpha = 0.001$] and effect size magnitudes (cf. Kitchenham et al. [49])

	PSP/LTL	PSP/EPL	EPL/LTL
Syntactic correctness			
$p_1 = P(X>Y)$	0.6303	0.4069	0.7718
$p_2 = P(X=Y)$	0.0058	0	0.006
$p_3 = P(X<Y)$	0.3697	0.5931	0.2222
*d*	$-0.2605$	0.1863	$-0.5496$
$s_d$	0.1923	0.1991	0.1404
*z*	$-1.3547$	0.9354	$-3.9153$
CI low	$-0.5429$	$-0.1513$	$-0.7397$
CI high	0.0748	0.4849	$-0.2783$
*p*	0.092	0.1777	0.0002
FDR adjusted *p*	0.1971	0.2961	0.0012
Level of significance	–	–	**
Effect size magnitude	–	–	Large
Semantic correctness			
$p_1 = P(X>Y)$	0.7815	0.4461	0.8373
$p_2 = P(X=Y)$	0	0.0025	0.002
$p_3 = P(X<Y)$	0.2185	0.5515	0.1607
*d*	$-0.563$	0.1054	$-0.6766$
$s_d$	0.1517	0.1938	0.1229
*z*	$-3.7109$	0.5438	$-5.5023$
CI low	$-0.7633$	$-0.2153$	$-0.8322$
CI high	$-0.2641$	0.4055	$-0.4221$
*p*	0.0003	0.2948	$9.6 \times 10^{-7}$
FDR adjusted *p*	0.0017	0.3641	$1.4 \times 10^{-5}$
Level of significance	**	–	***
Effect size magnitude	Large	–	Large
Response time			
$p_1 = P(X>Y)$	0.5686	0.4755	0.6349
$p_2 = P(X=Y)$	0.0112	0	0.002
$p_3 = P(X<Y)$	0.4202	0.5245	0.3631
*d*	$-0.1485$	0.049	$-0.2718$
$s_d$	0.194	0.1894	0.1697
*z*	$-0.7652$	0.2588	$-1.6016$
CI low	$-0.4451$	0.2595	$-0.5243$
CI high	0.1775	0.3485	0.0246
*p*	0.2246	0.3985	0.0583
FDR adjusted *p*	0.3062	0.4703	0.1507
Level of significance	–	–	–
Effect size magnitude	–	–	–

In the master-level course ASE (cf. Table 12), there is a large-sized difference in syntactic correctness between EPL and LTL. Regarding semantic correctness, there are large-sized effects between PSP/LTL and EPL/LTL, indicating that the former outperforms the latter mentioned approach. As in SE2, there are no significant differences regarding the response times. Consequently, $H_{0,1}$ can only be rejected on the basis of semantic correctness, whereas $H_{0,3}$ is rejected based on both types of correctness.

Table 13 contains the test results for participants with industry experience. There is no significant difference in terms of syntactic correctness and response time. Similarly to ASE, there is no significant difference in semantic correctness between PSP and EPL, while there are significant differences with large-sized effects when comparing PSP against LTL and EPL against LTL.

**Table 13:** Cliff’s *d* of syntactic/semantic correctness and response time for participants with industry experience $\ge 1$ year, one-tailed with confidence intervals calculated for $\alpha = 0.05$ (cf. Cliff [14] and Rogmann [70]), adjusted p-values (cf. Benjamini and Hochberg [8]) [level of significance: * for $\alpha = 0.05$, ** for $\alpha = 0.01$, *** for $\alpha = 0.001$] and effect size magnitudes (cf. Kitchenham et al. [49])

	PSP/LTL	PSP/EPL	EPL/LTL
Syntactic correctness			
$p_1 = P(X>Y)$	0.6471	0.5321	0.6636
$p_2 = P(X=Y)$	0.0029	0	0.0023
$p_3 = P(X<Y)$	0.35	0.4679	0.3341
*d*	$-0.2971$	$-0.0642$	$-0.3295$
$s_d$	0.1875	0.1934	0.1702
*z*	1.5845	$-0.3318$	$-1.9358$
CI low	$-0.5697$	$-0.3682$	$-0.5774$
CI high	0.0345	0.2523	$-0.0260$
*p*	0.061	0.371	0.0299
FDR adjusted *p*	0.1526	0.4752	0.1043
Level of significance	–	–	–
Effect size magnitude	–	–	–
Semantic correctness			
$p_1 = P(X>Y)$	0.7824	0.5802	0.7295
$p_2 = P(X=Y)$	0	0	0.0023
$p_3 = P(X<Y)$	0.2176	0.4198	0.2682
*d*	$-0.5647$	$-0.1604$	$-0.4614$
$s_d$	0.1501	0.1907	0.1582
*z*	$-3.7622$	$-0.8412$	$-2.9159$
CI low	$-0.7632$	$-0.4513$	$-0.6812$
CI high	$-0.2687$	0.1613	$-0.1652$
*p*	0.0003	0.2028	0.0029
FDR adjusted *p*	0.0023	0.3803	0.0145
Level of significance	**	–	*
Effect size magnitude	Large	–	Large
Response time			
$p_1 = P(X>Y)$	0.5059	0.5134	0.4909
$p_2 = P(X=Y)$	0.0029	0	0.0045
$p_3 = P(X<Y)$	0.4912	0.4866	0.5045
*d*	$-0.0147$	$-0.0267$	0.0136
$s_d$	0.1986	0.191	0.1822
*z*	$-0.074$	$-0.14$	0.0749
CI low	$-0.3314$	$-0.331$	$-0.2808$
CI high	0.305	0.2825	0.3057
*p*	0.4707	0.4447	0.4704
FDR adjusted p	0.4752	0.4752	0.4752
Level of significance	–	–	–
Effect size magnitude	–	–	–

**Table 14:** Cliff’s *d* of perceived correctness and ease of application in SE2 and ASE, one-tailed with confidence intervals calculated for $\alpha = 0.05$ (cf. Cliff [14] and Rogmann [70]), adjusted p-values (cf. Benjamini and Hochberg [8]) [level of significance: * for $\alpha = 0.05$, ** for $\alpha = 0.01$, *** for $\alpha = 0.001$] and effect size magnitudes (cf. Kitchenham et al. [49])

	PSP/LTL	PSP/EPL	EPL/LTL
*Software Engineering 2 (Bachelor-level course)*			
Perceived correctness			
$p_1 = P(X>Y)$	0.4336	0.4087	0.392
$p_2 = P(X=Y)$	0.2485	0.2589	0.259
$p_3 = P(X<Y)$	0.3179	0.3324	0.349
*d*	$-0.1157$	$-0.0763$	$-0.043$
$s_d$	0.05	0.0511	0.0502
*z*	$-2.3139$	$-1.494$	$-0.8566$
CI low	$-0.197$	$-0.1597$	$-0.1253$
CI high	$-0.0328$	0.0081	0.0398
*p*	0.0105	0.0679	0.196
FDR adjusted *p*	0.0316	0.1019	0.2262
Level of significance	*	–	–
Effect size magnitude	Medium	–	–
Perceived ease of application			
$p_1 = P(X>Y)$	0.4213	0.4005	0.3881
$p_2 = P(X=Y)$	0.2518	0.2569	0, 2631
$p_3 = P(X<Y)$	0.3269	0.3426	0.3488
*d*	$-0.0945$	$-0.0579$	$-0.0394$
$s_d$	0.0502	0.0513	0.0501
*z*	$-1.8827$	$-1.1272$	$-0.7857$
CI low	$-0.1762$	$-0.1417$	$-0.1214$
CI high	$-0.0114$	0.0268	0.0432
*p*	0.0302	0.1301	0.2162
FDR adjusted *p*	0.0658	0.1766	0.2317
Level of significance	–	–	–
Effect size magnitude	–	–	–
*Advanced Software Engineering (master-level course)*			
Perceived correctness			
$p_1 = P(X>Y)$	0.3675	0.4013	0.3095
$p_2 = P(X=Y)$	0.3039	0.2914	0.324
$p_3 = P(X<Y)$	0.3286	0.3074	0.3664
*d*	$-0.0389$	$-0.0939$	0.0569
$s_d$	0.0808	0.0778	0.0722
*z*	$-0.481$	$-1.2065$	0.7882
CI low	$-0.1706$	$-0.22$	$-0.0623$
CI high	0.0942	0.0352	0.1745
*p*	0.3155	0.1145	0.2157
FDR adjusted *p*	0.3641	0.2147	0.3062
Level of significance	–	–	–
Effect size magnitude	–	–	–
Perceived ease of application			
$p_1 = P(X>Y)$	0.4338	0.3752	0.4233
$p_2 = P(X=Y)$	0.2613	0.2616	0.2891
$p_3 = P(X<Y)$	0.3049	0.3632	0.2876
*d*	$-0.129$	$-0.012$	$-0.1356$
$s_d$	0.0827	0.0807	0.0725
*z*	$-1.5594$	$-0.1481$	$-1.8697$
CI low	$-0.262$	$-0.144$	$-0.2526$
CI high	0.009	0.1205	$-0.0147$
*p*	0.0603	0.4412	0.0314
FDR adjusted *p*	0.1507	0.4412	0.1178
Level of significance	–	–	–
Effect size magnitude	–	–	–

Tables 14 and 15 summarize the test results regarding perceived correctness and perceived ease of application. Almost all test results are not significant with two exceptions: (1) A significant test result ($p = 0.0316$) with a medium-sized effect is present in SE2 between PSP and LTL with regard to perceived correctness. Consequently, $H_{0,4}$ can be rejected in SE2. That is, PSP participants are significantly more confident that the formalization is correct than LTL participants at the bachelor level while such an effect is not measurable at the master level or within the sample of industry participants. (2) Participants with industry experience rate the ease of application of EPL significantly higher than of LTL ($p = 0.0023$). Consequently, $H_{0,9}$ can be rejected for participants with industry experience.

**Table 15:** Cliff’s *d* of perceived correctness and ease of application for participants with industry experience, one-tailed with confidence intervals calculated for $\alpha = 0.05$ (cf. Cliff [14] and Rogmann [70]), adjusted p-values (cf. Benjamini and Hochberg [8]) [Level of significance: * for $\alpha = 0.05$, ** for $\alpha = 0.01$, *** for $\alpha = 0.001$], and effect size magnitudes (cf. Kitchenham et al. [49])

	PSP/LTL	PSP/EPL	EPL/LTL
Perceived correctness			
$p_1 = P(X>Y)$	0.3586	0.344	0.37
$p_2 = P(X=Y)$	0.2778	0.2872	0.2745
$p_3 = P(X<Y)$	0.3636	0.3689	0.3555
*d*	0.0051	0.0249	$-0.0145$
$s_d$	0.0813	0.0793	0.0768
*z*	0.0622	0.3142	$-0.1894$
CI low	$-0.1283$	$-0.1055$	$-0.1402$
CI high	0.1383	0.1545	0.1116
*p*	0.4752	0.3769	0.425
FDR adjusted *p*	0.4752	0.4752	0.4752
Level of significance	–	–	–
Effect size magnitude	–	–	–
Perceived ease of application			
$p_1 = P(X>Y)$	0.4078	0.3006	0.5014
$p_2 = P(X=Y)$	0.2734	0.2524	0.2555
$p_3 = P(X<Y)$	0.3188	0.447	0.2432
*d*	$-0.0889$	0.1463	$-0.2581$
$s_d$	0.0826	0.0802	0.0733
*z*	$-1.0765$	1.8252	$-3.5228$
CI low	$-0.2226$	0.0124	$-0.3744$
CI high	0.048	0.2751	$-0.1339$
*p*	0.1416	0.0348	0.0003
FDR adjusted *p*	0.3033	0.1042	0.0023
Level of significance	–	–	**
Effect size magnitude	–	–	Medium

The statistics software *R*^7^ was used for all statistical analyses. In particular, the following libraries were used in the course of the performed statistical evaluations: *biotools* [75], *car* [33], *ggplot2* [85], *mvnormtest* [76], *mvoutlier* [63], *orddom* [70], *psych* [67] and *usdm* [58].

## Discussion

This sections discusses the results and threats to validity of the study.

### Evaluation of results and implications

The experimental goal was stated as ***Analyze****LTL, PSP and EPL****for the purpose of****their evaluation****with respect to****their understandability related to modeling compliance specifications****from the viewpoint of****the novice and moderately advanced software engineer, designer or developer****in the context/environment of****the Software Engineering 2 Lab and the Advanced Software Engineering Lab courses at the Faculty of Computer Science of the University of Vienna.* Due to the large number of participants with industry experience, it became possible to consider a third population, namely participants with industry experience, who function as proxies for industrial practitioners with basic to modest industry experience. Based upon the stated goal, questions concerning *understandability* were generated. The understandability construct focuses on the degree of syntactic and semantic correctness achieved and on the time spent on modeling compliance specifications. The results per question are summarized in Table 16. By differentiating between syntactic and semantic correctness, it became possible to reveal that differences in understandability in formal modeling of compliance specifications predominately lie in semantic correctness. Almost all test results regarding semantic correctness are highly significant with large-sized effects. Interestingly, no significant difference in semantic correctness is present between the pattern-based PSP approach and the CEP-based EPL language in the master-level course ASE and in the subset of participants with industry experience. That might imply that more experienced users are able to cope equally well with both approaches. Aside from that, the results suggest that the pattern-based PSP approach is more understandable than EPL and LTL and that EPL provides a higher level of understandability than LTL. In terms of syntactic correctness, PSP seems to be more understandable than LTL for less experience users, while EPL seems to be more understandable than LTL for more experienced users. This study did not reveal any significant differences in response time. Regarding perceived correctness and perceived ease of application, there are two significant test results, which imply that transformations to PSP are perceived to be more correct than LTL transformations by less experienced users, and more experienced users with industry experience find that EPL is easier to apply than LTL.

Overall, the results imply that the pattern-based PSP approach has advantages with regard to understandability. Therefore, the pattern-based approach seems to be particularly well suited for modeling compliance specifications. Moreover, the results indicate that EPL is more understandable than LTL. This could be important in cases where the set of available PSP patterns is not sufficient to model a compliance specification. In such cases, the compliance specification could be encoded in EPL for runtime verification or an extension of the pattern catalog could take place. In this regard, EPL specifications could be used to aid the creation of new patterns with underlying LTL formalizations by checking the plausibility of the LTL formula (cf. Czepa et al. [18, 19]).

Moreover, the results are overall in line with two controlled experiments on the understandability of already existing formal specifications in LTL, EPL and PSP carried out by Czepa and Zdun [17]. The results of these controlled experiments with 216 participants in total suggested that existing specifications in PSP are significantly easier to understand than existing specifications in EPL and LTL. Moreover, the results implied that existing specifications in EPL are significantly easier to understand than existing specifications in LTL. The correctness of understanding was evaluated by letting the participant decide whether a truth value is the correct truth value of a specification, given a specific trace. In contrast to the current study, which focuses on the formal modeling of compliance specifications, no major differences between novice and moderately advanced users were found in understandability of existing specifications. Interestingly, the response times between the experimental groups were significantly different in most cases, an effect which appears to be absent during modeling (cf. Sect. 4.3).

**Table 16:** GQM summary

ID	Question	Summary of results
Q1	How understandable are the tested approaches for participants at the bachelor level (attending the Software Engineering 2 Lab course)?	
Q2	Are there differences in understandability between the tested approaches for participants at the bachelor level (attending the Software Engineering 2 Lab course)?	There are significant differences between all tested approaches in terms of semantic correctness, and between PSP and LTL in terms of syntactic correctness
Q3	How understandable are the tested approaches for participants at the master level (attending the Advanced Software Engineering Lab course)?	
Q4	Are there differences in understandability between the tested approaches for participants at the master level (attending the Advanced Software Engineering Lab course)?	There are significant differences in terms of semantic and syntactic correctness between EPL and LTL, and between PSP and LTL in terms of semantic correctness
Q5	How understandable are the tested approaches for participants with industrial working experience?	
Q6	Are there differences in understandability between the tested approaches for participants with industrial working experience?	There are significant differences in terms of semantic correctness between PSP and LTL as well as between EPL and LTL

### Threats to validity

In the following, all known threats that might have an impact on the validity of the results of this study are discussed.

#### Threats to internal validity

Threats to internal validity are unobserved variables that might have an undesired impact on the result of the experiment by disturbing the causal relationship of independent and dependent variables. There exist several threats to internal validity, which must be discussed:

- *History effects* refer to events that happen in the environment resulting in changes in the conditions of a study. The short duration of the study limits the possibility of changes in environmental conditions, and none were observed. The occurrence of such effects prior to the study cannot be entirely ruled out. However, in such a case, it would be extremely unlikely that the scores of one experiment group are more affected than another, because of the random allocation of participants to groups.
- *Maturation effects* refer to the impact the passage of time has on an individual. Like history effects, maturation effects are rather problematic in long-term studies. Since the duration of the experiment was short, maturation effects are considered to be of minor importance, and none were observed.
- *Testing effects* comprise learning effects and experimental fatigue. *Learning effects* were avoided by testing each person only once. *Experimental fatigue* is concerned with happenings during the experiment that exhaust the participant either physically or mentally. The short time frame of the experiment session limits chances of fatigue. Neither were any signs of fatigue observed nor were there any reports by participants indicating fatigue.
- *Instrumental bias* occurs if the measuring instrument (i.e., a physical measuring device or the actions/assessment of the researcher) changes over time during the experiment. Since the answers given in the experiment tasks were evaluated manually, this is a serious threat to validity. It might be the case that the experience gained in scoring some answers had an influence on subsequent evaluations. This threat was mitigated by evaluating the results in no specific prescribed order, and in case of substantial differences in grading, a discussion took place until consensus was achieved.
- *Selection bias* is present if the experimental groups are unequal before the start of the experiment (e.g., severe differences in previous experience). Selection bias is likely to be more threatening in quasi-experimental research. By using an experimental design with the fundamental requirement to randomly assignment participants to the different groups of the experiment, it became possible to avoid selection bias to a large extent. In addition, the investigation of the composition of the groups did not reveal any major differences between them. (cf. “Appendix D”).
- *Experimental mortality* more likely occurs in long-lasting experiment since the chances for dropouts increase (e.g., participants leaving the town). Due to the short time frame of this study, experimental mortality did not occur.
- *Diffusion of treatments* is present if at least one group is contaminated by the treatments of at least one other group. Since the participants share the same social group, and they are interacting outside the research process as well, a cross-contamination between the groups cannot be entirely rule out.
- *Compensatory rivalry* is present if participants of a group put in extra effort when the impression arises that the treatment of another group might lead to better results than their own treatment. This threat was mitigated by clarifying that different degrees of difficulty will be considered and compensated in the calculation of bonus points.
- *Demoralization* could occur if a participant is assigned to a specific group that she/he does not want to be part of. No indications of demoralization such as increased dropout rates or complaints regarding group allocation were observed.
- *Experimenter bias* refers to undesired effects on the dependent variables that are unintentionally introduced by the researcher. All participants received a similar training and worked on the same set of tasks. A manual evaluation of the given answers regarding their correctness was performed. To mitigate the threat of experimenter bias in that regard, the first, second and third author performed the evaluation of all tasks individually. Differentiating between semantic and syntactic correctness overall simplified the evaluation process by enabling a separation of concerns. A potential threat in that regard could be falsely classifying defects. Therefore, after the completion of all individual evaluations, in case of substantial differences in grading, a discussion took place until consensus was achieved.

#### Threats to external validity

The external validity of a study focuses on its generalizability. In the following, potential threats that hinder a generalization are discussed. Different types of generalizations must be considered:

- *Generalizations across populations*: By statistical inference, generalizations from the sample to the immediate population are made. The initial study design considered two populations, namely computer science students that enrolled in the course SE2 as proxies for novice software engineers, designers or developers, as well as computer science students that enrolled in the course ASE as proxies for moderately advanced software engineers, designers or developers. Due to the large number of participants with industry experience, it became possible to consider a third population, namely participants with industry experience, who function as proxies for industrial practitioners with basic to modest industry experience. The results of this study show interesting discrepancies between these populations. In particular, there are no significant differences in understandability between PSP and EPL for more advanced users while a significant difference is measurable when testing less experienced users. In general, this study does not intent to claim generalizability to other populations without further empirical evidence. For example, it might be plausible that leading experts working in the software industry or as business administrators perform similarly to ASE participants or the subset of participants with industry experience, but this study can neither support nor reject such claims.
- *Generalizations across treatments*: The treatments are equivalent to specific tested languages. Treatment variations would likely be related to changing the contents, amount or difficulty of experiment tasks or the amount of training provided. The experiment design attempts to be as general as possible by using compliance specifications stemming from different domains and applying a moderate amount of training.
- *Generalizations across settings/contexts*: The participants of this study are students who enrolled computer science courses at the University of Vienna, Austria. The majority of the students are Austrian citizens, but there is a large presence of foreign students as well. Surely, it would be interesting to repeat the experiment in different settings/context to evaluate the generalizability in that regard. For example, repeating the experiment with English native speakers might lead to different and presumably better results.
- *Generalizations across time*: It is hard to foresee whether the results of this study will hold over time. For example, if teaching of a specific tested language is intensified in the computer science curricula at the University of Vienna, then the students would bring in more expertise, which likely would have an impact on the results.

#### Threats to construct validity

There are potential threats to the validity of the construct that must be discussed:

- *Inexact definition and Construct confounding*: This study has a primary focus on the construct *understandability*, which is measured by the dependent variables *syntactic correctness*, *semantic correctness* and *response time*. This construct is exact and adequate, and the dependent variables *syntactic correctness* and *semantic correctness* make even a more fine-grained analysis possible than in existing studies that measure correctness by a single variable (cf. Feigenspan et al. [31] and Hoisl et al. [40]).
- *Mono-method bias*: Due to organizational reasons, keeping time records was the personal responsibility of each participant. The participants were carefully instructed how to record start and end times, and we did not detect any irregularities (e.g., overlapping time frames or long pauses) in those records. Nonetheless, this measuring method leaves room for measuring errors, and an additional or alternative measuring method (e.g., direct observation by experimenters or performing the experiment with an online tool that handles record keeping) would reduce this threat. However, these methods would have influenced the overall study design and potentially could have introduced other threats to validity (e.g., prolonged experiment execution potentially leading to an exposure of the experiment task contents or technical problems during experiment execution). To avoid mono-method bias in evaluating the syntactic and semantic correctness, the grading was not performed by a single but by three experimenters individually.
- *Reducing levels of measurements*: Both correctness variables and the response time are continuous variables. That is, the levels of measurements are not reduced. The Likert scales used in this study offer 5 answer categories rather than 7 or 11, because the latter mentioned would produce data of lower quality according to Revilla et al. [68].
- *Treatment-sensitive factorial structure*: In some empirical studies, a treatment might sensitize participants to develop a different view on a construct. The actual level of understandability based on the task solutions provided was measured, so the participants’ view on this construct appears to be irrelevant.

#### Threats to content validity

Content validity is concerned with the relevance and representativeness of the elements of a study for the measured construct:

- *Relevance*: The tasks of this study are based on realistic scenarios stemming from three different domains in which compliance is highly relevant (cf. Elgammal et al. [29], Rovani et al. [71], and United States Environmental Protection Agency [83]).
- *Representativeness*: In the formal modeling of the compliance specifications, the use of all core temporal LTL operators and EPL operators was required, which means that the construct understandability was measured comprehensively. The use of each PSP pattern was required two or more times (cf. sample solutions of experimental tasks in “Appendix A”). Unfortunately, it was not possible to test all available pattern–scope combinations. However, the majority of specifications are based on the *global* scope (cf. Dwyer et al. [27, 28]), which is as well reflected in the realistic specifications used in the tasks of this experiment (cf. experimental tasks in Table 7 and sample solutions in “Appendix A”). That is, a representative subset of PSP was tested.

#### Threats to conclusion validity

Thorough statistical investigations of model assumptions were performed before applying the most suitable statistical test with the greatest statistical power, given the properties of the acquired data. That course of action is considered to be highly beneficial to the conclusion validity of this study. The decision to retain outliers might be a threat to conclusion validity, but all outliers appear to be valid measurements, so deleting them would pose a threat to conclusion validity as well.

## Related work

We are not aware of any empirical studies evaluating the understandability related to the formal modeling of compliance specifications in particular. There exists, however, related work focusing on similar issues.

Related studies in the field of business process management are concerned with declarative workflows (cf. van der Aalst [1]), which use graphical patterns with underlying formal representations in LTL (cf. Montali [56]) or event calculus (cf. Montali et al. [57]). Haisjackl and Zugal [35] investigated differences between textual and graphical declarative workflows in an empirical study with 9 participants. The descriptive statistics of this study indicates that the graphical representation is advantageous in terms of perceived understandability, error rate, duration and mental effort. The lack of hypothesis testing and the small number of participants are severe threats to the validity of this study. Zugal et al. [87] investigated the understandability of hierarchies on basis of the same data set. The results of their research indicate that hierarchies must be handled with care. While information hiding and improved pattern recognition are considered to be positive aspects of hierarchies since the mental effort for understanding a process model is lowered, the fragmentation of processes by hierarchies might lower overall understandability of the process model. Another important finding of their study is that users appear to approach declarative process models in a sequential manner even if the user is definitely not biased by previous experiences with sequential/imperative business process models. They conclude that the abstract nature of declarative process models does not seem to fit the human way of thinking. Moreover, they observed that the participants of their study tried to reduce the number of constraints to consider by putting away sheets that describe irrelevant sub-process or by using the hand to hide parts of the process model that are irrelevant. Like in the previously discussed study, it must be assumed that the validity of this study is strongly limited by the extremely small sample size. Haisjackl et al. [36] investigate the users’ understanding of declarative business process models, again on the same data set. As in the previously mentioned study, they point out that users tend to read such models sequentially despite the declarative nature of the approach. The larger a model, the often are hidden dependencies overlooked, which indicates increasing numbers of constraints lower understanding. Moreover, they report that single constraints are overall well understood, but there seem to be problems with understanding the precedence constraint. As the authors point out, this kind of confusion could be related to the graphical arrow-based representation of the constraints where subtle differences decide on the actual meaning. That is, the arrow could be confused with a sequence flow as present in flow-driven, sequential business processes. As previously stated for the other two studies that are based on the same data set, the validity of this study is possibly strongly affected by the small sample size. De Smedt et al. [26] tried to improve the understandability of declarative business process models by explicitly revealing hidden dependencies. They conduced an experiment with 95 students. The result suggests that explicitly showing hidden dependencies enables a better understandability of declarative business process models. Pichler et al. [64] compared the understandability of imperative and declarative business process modeling notations. The results of this study are in line with Zugal et al. [87] and suggest that imperative process models are significantly better understandable than declarative models, but the authors also state that the participants had more previous experience with imperative process modeling than with declarative process modeling. The small sample size (28 participants) is a threat to validity of this study. Rodrigues et al. [69] compared the understandability of textual and graphical BPMN [59] business process descriptions with 32 students and 41 practitioners. They conclude that experienced users understand a process better if it is presented by a graphical BPMN process model whereas for inexperienced users there is no difference in understandability between the textual and graphical process descriptions. Jost et al. [46] compared the intuitive understanding of process diagrams with 103 students. They conclude that UML activity diagrams provide a higher level of understandability than BPMN diagrams and EPCs.

Software architecture compliance, which focuses on the alignment of software architecture and implementation, and requirements engineering are also related to this study. Czepa et al. [21] compared the understandability of three languages for behavioral software architecture compliance checking, namely the natural language constraint (NLC) language, the cause–effect constraint (CEC) language and the temporal logic pattern-based constraint (TLC) language, in a controlled experiment with 190 participants. The NLC language is simply referring to using the English language for documenting software architectures. CEC is a high-level structured architectural description language that abstracts EPL. It supports the nesting of cause parts, which observe an event stream for a specific event pattern, and effect parts, which can contain further cause–effect structures and truth value change commands. TLC is a high-level structured architectural description language based on PSP. Interestingly, the statistical inference of this study suggests that there is no difference in understandability of the tested languages. This could indicate that the high-level abstractions employed bring those structured languages closer to the understandability of unstructured natural language architecture descriptions. Moreover, it might also suggest that natural language leaves more room for ambiguity, which is detrimental for its understanding. Potential limitations of that study are that its tasks are based on common architectural patterns/styles (i.e., a participant possibly recognizes the meaning of a constraint more easily by having knowledge of the related architectural pattern) and the rather small set of involved patterns (i.e., only very few patterns of PSP were necessary to represent the architecture descriptions). A controlled experiment carried out by Heijstek et al. [38] with 47 participants focused on finding differences in understanding of textual and graphical software architecture descriptions. Interestingly, participants who predominantly used textual architecture descriptions performed significantly better, which suggests that textual architectural descriptions could be superior to their graphical counterparts. An eye-tracking experiment with 28 participants by Sharafi et al. [74] on the understandability of graphical and textual software requirement models did not reveal any statistically significant difference in terms of correctness of the approaches. The study also reports that the response times of participants working with the graphical representations were slower. Interestingly though, the participants preferred the graphical notation. Hoisl et al. [40] conducted a controlled experiment on three notations for scenario-based model tests with 20 participants. In particular, they evaluated the understandability of a semi-structured natural language scenario notation, a diagrammatic scenario notation and a fully structured textual scenario notation. According to the authors, the purely textual semi-structured natural language scenario notation is recommended for scenario-based model tests, because the participants of this group were able to solve the given tasks faster and more correctly. That is, the study might indicate that a textual approach outperforms a graphical one for scenario-based model test, but the validity of the experiment is limited by the small sample size and the absence of statistical hypothesis testing.

## Conclusion and future work

The main goal of this empirical study was testing and comparing the understandability of representative approaches for the formal modeling of compliance specifications. The experiment was conducted with 215 participants in total. Major differences were found especially in semantic correctness of the approaches. Since formalizations in the property specification patterns (PSP) were overall more correct than in linear temporal logic (LTL) and event processing language (EPL), there is evidence that the pattern-based PSP approach provides a higher level of understandability. More advanced users, however, seemingly are able to cope equally well with PSP and EPL. That is, for more advanced users, these approaches can be used interchangeably as fitting best to a concrete domain or task. Moreover, EPL provides a higher level of understandability than LTL. Therefore, EPL is well suitable in situations that demand runtime verification in which the set of available patterns in PSP is not sufficient to model a compliance specification or to aid the creation of new patterns with underlying LTL formalizations (cf. Czepa et al. [18, 19]).

Moreover, the results are overall in line with two controlled experiments with 216 participants in total on the understandability of already existing formal specifications in LTL, EPL and PSP (cf. Czepa and Zdun [16]). In contrast to the current study, which focuses on the formal modeling of compliance specifications, no major differences between novice and moderately advanced users were found in understandability of existing specifications. Interestingly, the response times between the experimental groups were significantly different in most cases, an effect which appears to be absent during modeling.

Opportunities for further empirical research are the consideration of an extended set of representations including, for example, event calculus (cf. Kowalski and Sergot [52]) or Declare (cf. Pešić and van der Aalst [61]) and studying the understandability construct in different settings with other user groups (e.g., business administrators or professional software engineers). Moreover, besides the understandability construct, additional metrics such as changeability (i.e., “Is one representation easier to change when taking new/amended compliance specifications into account?”) and verifiability (i.e., “Are there differences between the representations when it comes to assessing whether a given compliance specification is fully covered?”) could be investigated.

## Figures and Tables

**Fig. 7:**
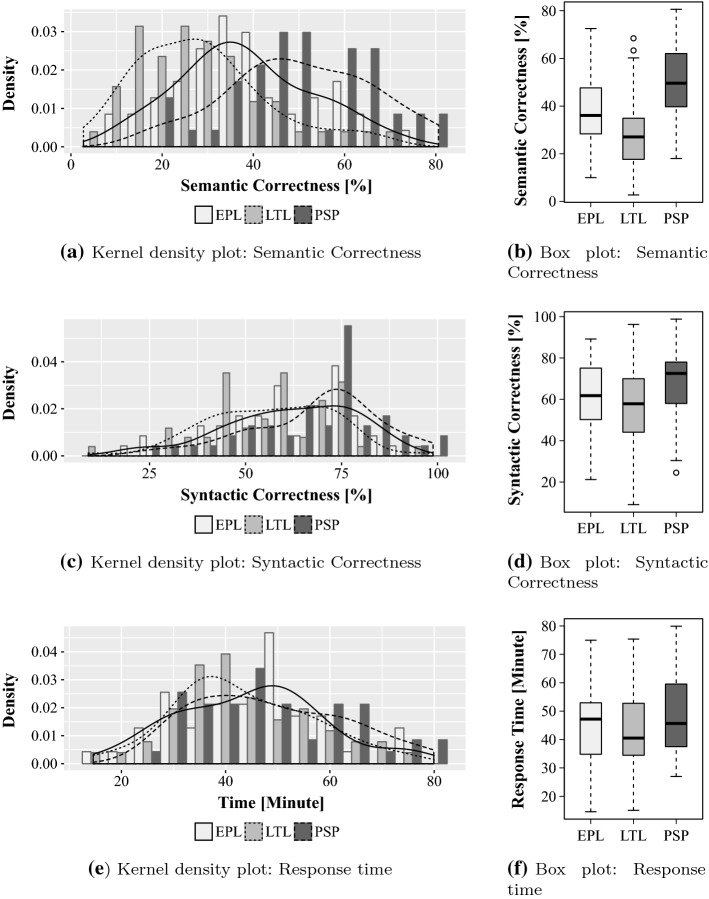
Kernel density plots and box plots of the participants’ semantic/syntactic correctness and response time of the given answers per group in the Software Engineering 2 (bachelor-level) course

**Fig. 8:**
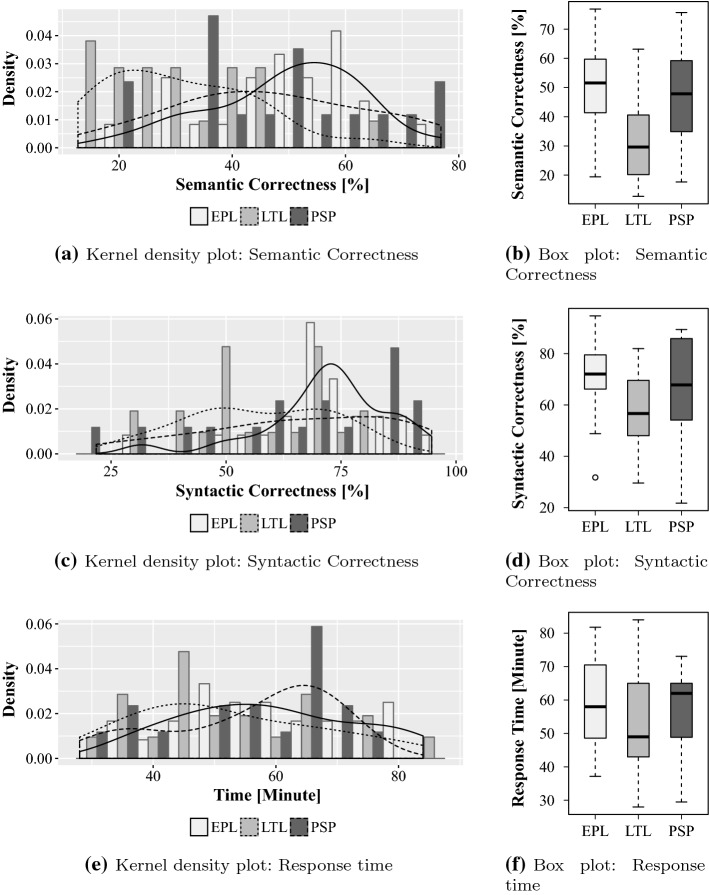
Kernel density plots and box plots of the participants’ semantic/syntactic correctness and response time of the given answers per group in the Advanced Software Engineering (master-level) course

**Fig. 9:**
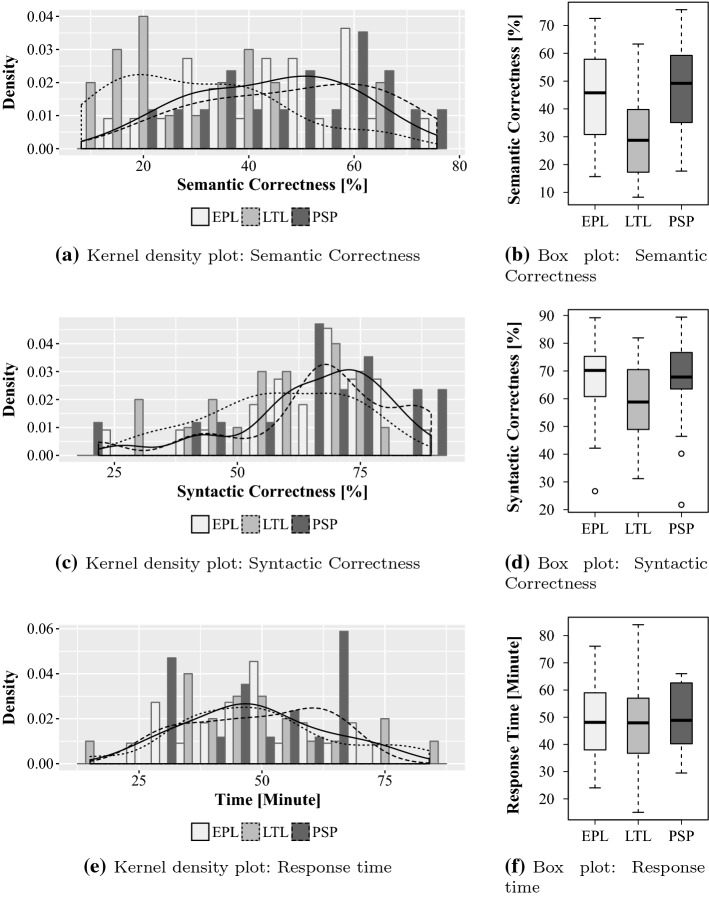
Kernel density plots and box plots of the participants’ semantic/syntactic correctness and response time of the given answers per experiment group of participants with $\ge 1$ year industry experience

**Fig. 10:**
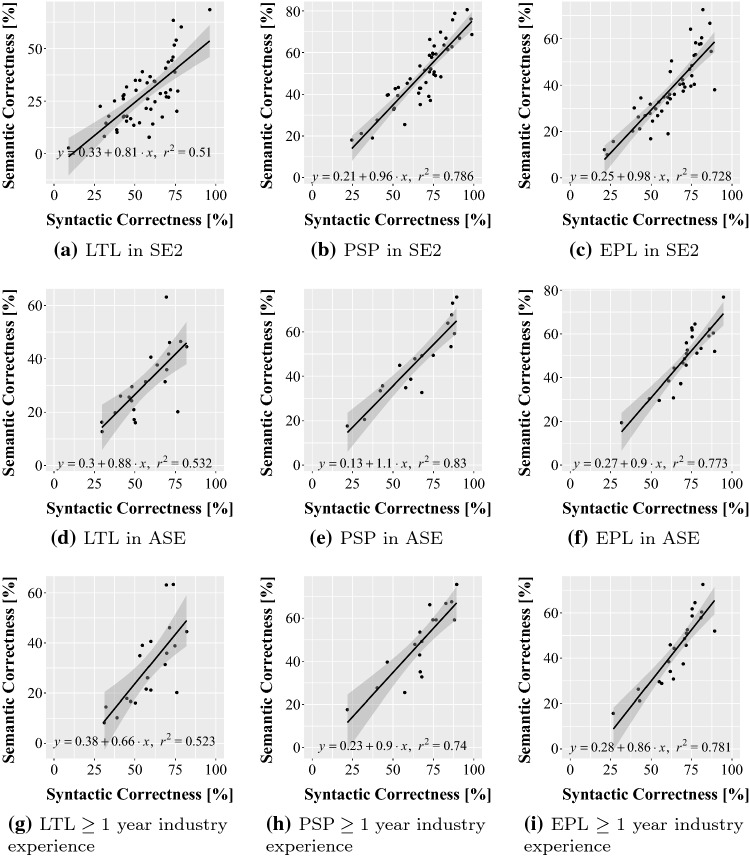
Scatter plots of syntactic vs. semantic correctness with linear trend lines, 95% confidence regions and coefficients of determination ($r^2$)

**Fig. 11:**
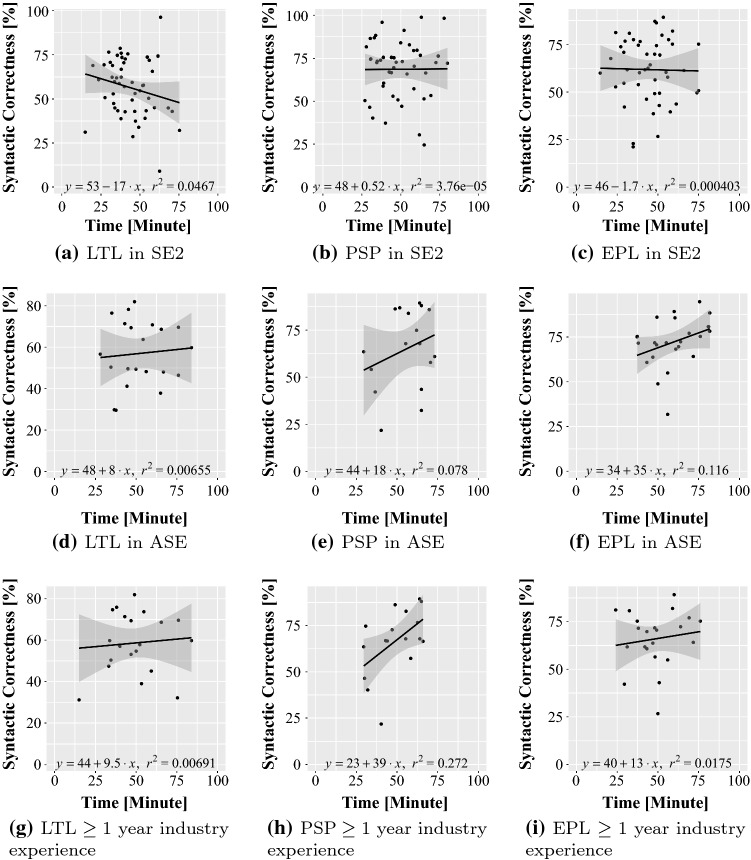
Scatter plots of time vs. syntactic correctness with linear trend lines, 95% confidence regions and coefficients of determination ($r^2$)

**Fig. 12:**
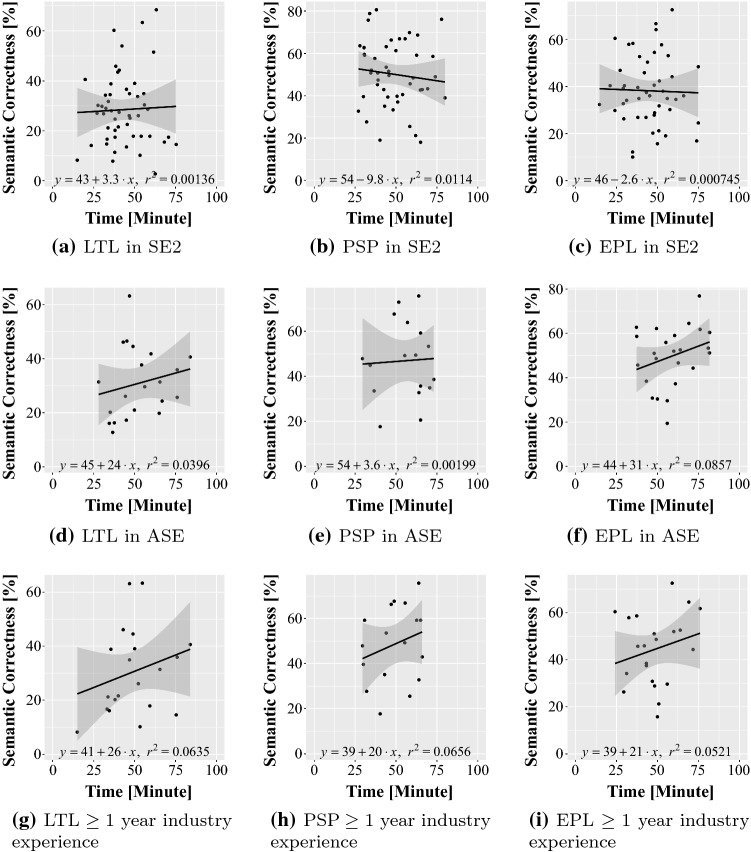
Scatter plots of time vs. semantic correctness with linear trend lines, 95% confidence regions and coefficients of determination ($r^2$)

**Fig. 13:**
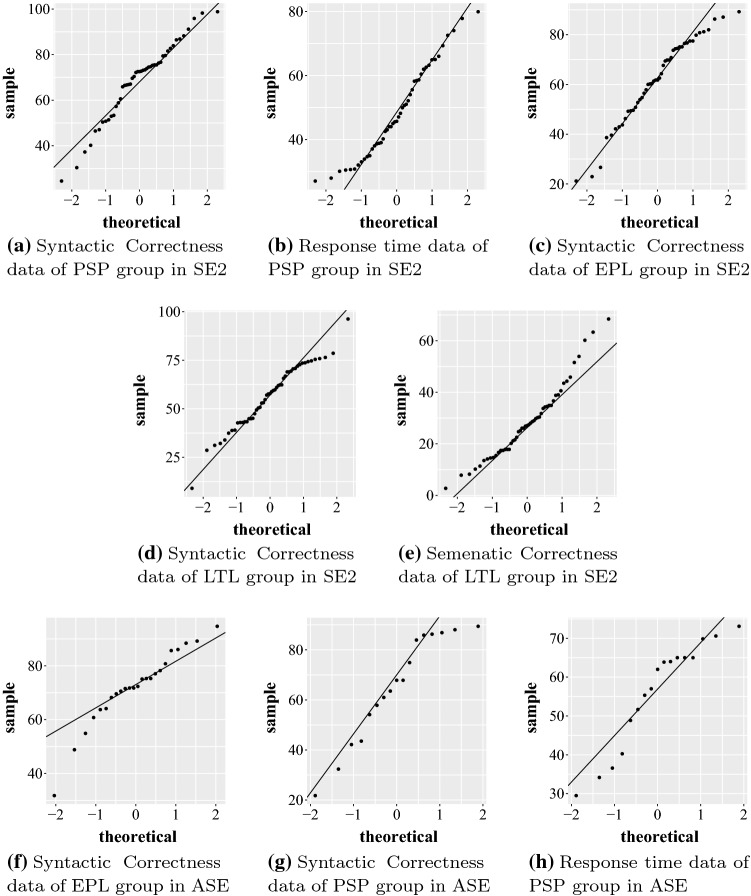
Normal QQ plots

**Fig. 14:**
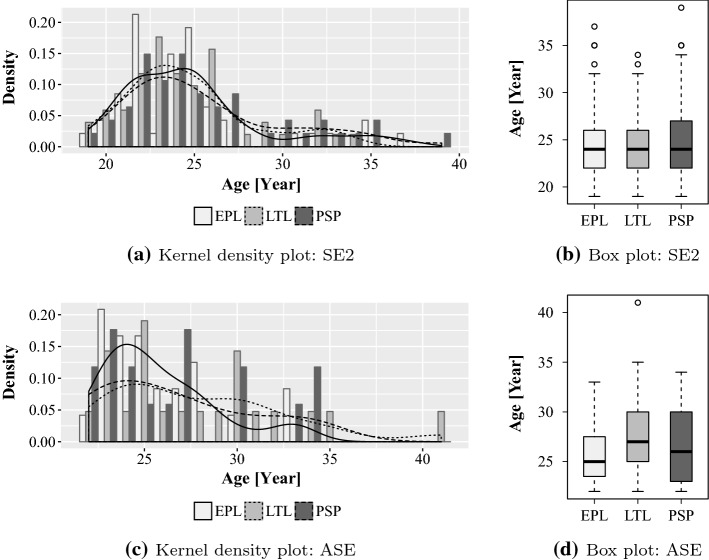
Kernel density plots and box plots of the participants’ age per group and course

**Fig. 15:**
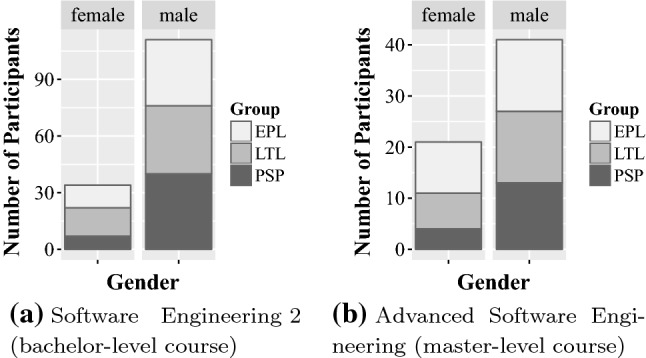
Bar charts of the participants’ gender per group and course

**Fig. 16:**
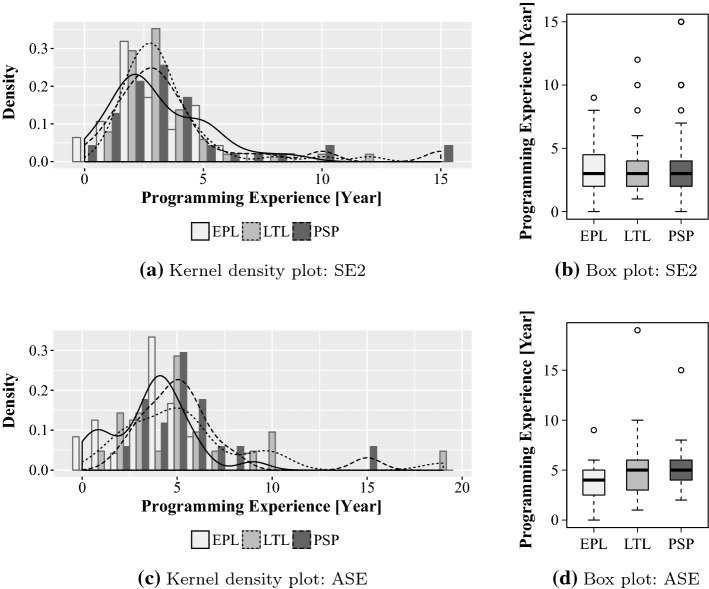
Kernel density plots and box plots of the participants’ programming experience per group and course

**Fig. 17:**
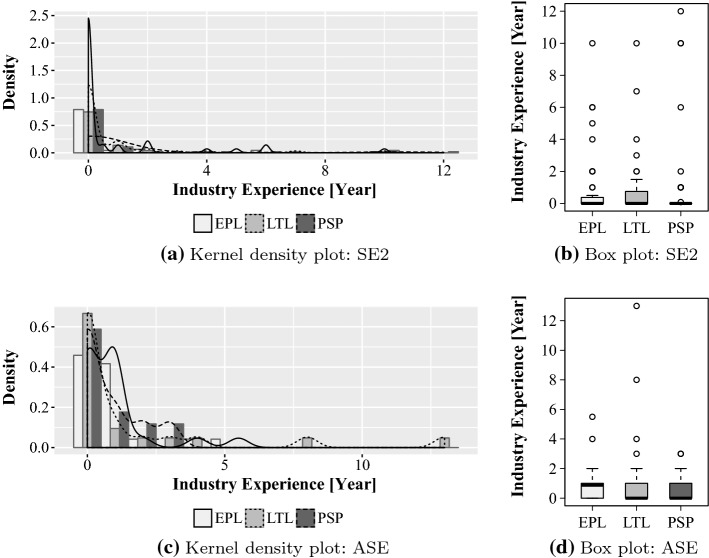
Kernel density plots and box plots of the participants’ software industry work experience per group and course

**Fig. 18:**
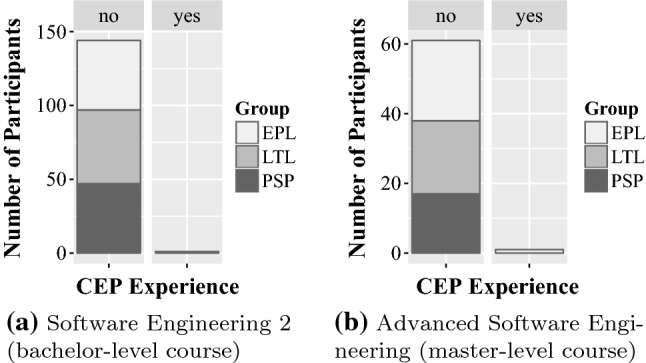
Bar charts of the participants’ prior experience with Complex Event Processing per group and course

**Fig. 19:**
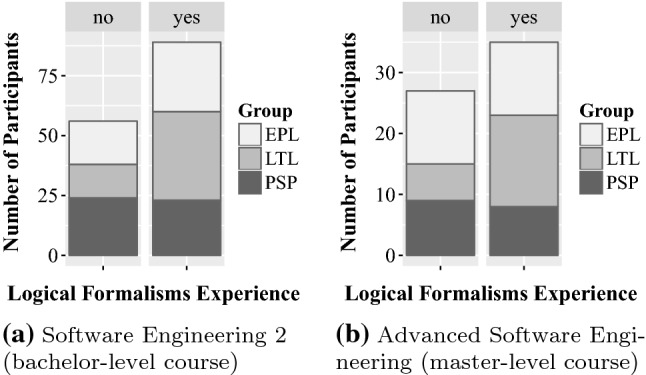
Bar charts of the participants’ prior experience with logical formalisms per group and course

## Appendix A: Sample solutions of experimental tasks

See Tables 17, 18, 19, 20 and 21.

**Table 17:** Sample solution of Task 1

Group	Sample solution
EPL	init ==> TS
	not ‘Evaluate Loan Risk’.completed until ‘Officially Sign Contract’.started ==> PV
	‘Evaluate Loan Risk’.completed ==> PS
	init ==> TS
	‘Evaluate Loan Risk’.role != ‘Branch Office Manager’ ==> PV
	init ==> TS
	‘Officially Sign Contract’.role != ‘Branch Office Manager’ ==> PV
LTL	! ‘Officially Sign Contract’.started W ‘Evaluate Loan Risk’.completed
	G! (‘Officially Sign Contract’.role != ‘Branch Office Manager’)
	G! (‘Evaluate Loan Risk’.role != ‘Branch Office Manager’)
PSP	‘Evaluate Loan Risk’.completed precedes ‘Officially Sign Contract’.started
	‘Officially Sign Contract’.role != ‘Branch Office Manager’ never occurs
	‘Evaluate Loan Risk’.role != ‘Branch Office Manager’ never occurs

**Table 18:** Sample solution of Task 2

Group	Sample solution
EPL	init ==> TS
	every(‘Check Customer Privilege’.completed -> ‘Check Credit Worthiness’.started) ==> TS
	every ‘Check Customer Privilege’.started ==> TV
	init ==> TS
	not ‘Check Customer Privilege’.completed until ‘Evaluate Loan Risk’.started ==> PV
	‘Check Customer Privilege’.completed ==> PS
	init ==> TS
	not ‘Check Credit Worthiness’.completed until ‘Evaluate Loan Risk’.started ==> PV
	‘Check Credit Worthiness’.completed ==> PS
LTL	G(‘Check Customer Privilege’.completed -> F ‘Check Credit Worthiness’.started)
	! ‘Evaluate Loan Risk’.started W ‘Check Customer Privilege’.completed
	! ‘Evaluate Loan Risk’.started W ‘Check Credit Worthiness’.completed
PSP	‘Check Customer Privilege’.completed leads-to ‘Check Credit Worthiness’.started
	‘Check Customer Privilege’.completed precedes ‘Evaluate Loan Risk’.started
	‘Check Credit Worthiness’.completed precedes ‘Evaluate Loan Risk’.started

**Table 19:** Sample solution of Task 3

Group	Sample solution
EPL	init ==> TS
	not ‘Preoperative Screening’.completed until ‘Laparoscopic Gastrectomy’.started ==> PV
	‘Preoperative Screening’.completed ==> PS
	init ==> TS
	not ‘Preoperative Screening’.completed until ‘Open Gastrectomy’.started ==> PV
	‘Preoperative Screening’.completed ==> PS
	init ==> TS
	‘Open Gastrectomy’.started leads-to ‘Laparoscopic Gastrectomy’.started ==> PV
	‘Laparoscopic Gastrectomy’.started leads-to ‘Open Gastrectomy’.started ==> PV
	init ==> TS
	every(‘Laparoscopic Gastrectomy’.completed leads-to ‘Nursing’.started) ==> TS
	every ‘Laparoscopic Gastrectomy’.completed ==> TV
	init ==> TS
	every(‘Open Gastrectomy’.completed leads-to ‘Nursing’.started) ==> TS
	every ‘Open Gastrectomy’.completed ==> TV
LTL	! ‘Laparoscopic Gastrectomy’.started W ‘Preoperative Screening’.completed
	! ‘Open Gastrectomy’.started W ‘Preoperative Screening’.completed
	(F ‘Open Gastrectomy’.started -> G! ‘Laparoscopic Gastrectomy’.started) & (F ‘Laparoscopic Gastrectomy’.started -> G! ‘Open Gastrectomy’.started)
	G(‘Laparoscopic Gastrectomy’.completed -> F ‘Nursing’.started)
	G(‘Open Gastrectomy’.completed -> F ‘Nursing’.started)
PSP	‘Preoperative Screening’.completed precedes ‘Laparoscopic Gastrectomy’.started
	‘Preoperative Screening’.completed precedes ‘Open Gastrectomy’.started
	after ‘Open Gastrectomy’.started [ ‘Laparoscopic Gastrectomy’.started never occurs ]
	after ‘Laparoscopic Gastrectomy’.started [ ‘Open Gastrectomy’.started never occurs ]
	‘Laparoscopic Gastrectomy’.completed leads-to ‘Nursing’.started
	‘Open Gastrectomy’.completed leads-to ‘Nursing’.started

**Table 20:** Sample solution of Task 4

Group	Sample solution
EPL	init ==> TS
	every(‘Lead Contamination identified’ leads-to not ‘Renovation’.completed until [‘Cleaning’.running and not ‘Presence of Certified Renovator’.running]) ==> PV
LTL	G(‘Lead Contamination identified’ & ! ‘Renovation’.completed -> (! (‘Cleaning’.running & ! ‘Presence of Certified Renovator’.running) W ‘Renovation’.completed))
PSP	after ‘Lead Contamination identified’ until ‘Renovation’.completed [‘Cleaning’.running and not ‘Presence of Certified Renovator’.running never occurs]

**Table 21:** Sample solution of Task 5

Group	Sample solution
EPL	init ==> TS
	not b.finished until r.started ==> PV
	not r.started until b.finished ==> PS
	init ==> TS
	not d.finished until r.started ==> PV
	not r.started until d.finished ==> PS
	init ==> TS
	not p.started until [y $\texttt {<}$ 1978 and (t = ‘residential house’ or t = ‘apartment’ or t = ‘child-occupied facility’) and renovation.started] ==> PV
	p.started ==> PS
LTL	!r.started W (b.finished & !r.started)
	!r.started W (d.finished & !r.started)
	!(y $\texttt {<}$ 1978 & (t = ‘residential house’ | t = ‘apartment’ | t = ‘child-occupied facility’) & renovation.started)) W p.started
PSP	before r.started [ b.finished occurs ]
	before r.started [ d.finished occurs ]
	p.started precedes (y $\texttt {<}$ 1978 and (t = ‘residential house’ or t = ‘apartment’ or t = ‘child-occupied facility’) and renovation.started)

## Appendix B: Extended descriptive statistics of dependent variables

Figure 7 shows kernel density plots and box plots of the dependent variables *syntactic correctness*, *semantic correctness* and *response time* in the SE2 course. As the kernel density plot in Fig. 7a clearly indicates, there are differences in central location and shape between the semantic correctness distributions of the groups. While the LTL group has a very low density in the range of 50–100 % semantic correctness, the PSP has a high density in the range of 40–70 % semantic correctness. The central location of the EPL group (about $35 \%$ semantic correctness) is located between the peaks of the two other distributions. Figure 7b shows two outliers in the LTL group, which represent participants who were able to achieve a higher level of correctness than most of their colleagues in the same experiment group. In Fig. 7c, a kernel density plot of the syntactic correctness is shown. All distributions have their central location at 70–75 min, but their shapes are different. The PSP distribution has a particularly high density directly at the central location whereas the remaining distributions show higher densities in the lower correctness ranges. There is a single outlier in the PSP group indicating a participant who has achieved a slightly lower level of syntactic correctness (cf. Fig. 7d). Both the kernel density plot in Fig. 7c and the box plot in Fig. 7d indicate a clear difference in distribution. The assumption of equal variance seems violated. The same applies to the response time distributions shown in Fig. 7e, f.

Figure 8 visualizes the data of the dependent variables *syntactic correctness*, *semantic correctness* and *response time* of ASE participants by kernel density plots and box plots. In Fig. 8a, The PSP semantic correctness distribution is rather flat with its peak at about $45 \%$. While the LTL semantic correctness distribution has a high density in the lower correctness range (10–45 %) with its peak density at 20–25 %, the EPL distribution has a high density in the range of 45–65 %. Thus, all semantic correctness distributions appear to be different in shape and central location. Regarding syntactic correctness (cf. Fig. 8c), the LTL distribution appears to be bimodal with peaks at $50 \%$ and $70 \%$. The EPL syntactic correctness distribution is steeper than the others with its peak at 70–75 %. In contrast, the PSP syntactic correctness distribution is strikingly flat with a slightly higher density in the higher syntactic correctness ranges. There is a single outlier in the EPL group showing a low level of syntactic correctness. The PSP group has its peak response time density at 65 min, and there are indications of bimodality with a second small peak at about 35 min. Both remaining response time distributions are rather similar of shape, but their central locations differ. LTL has its central location at 45 min whereas PSP has it at 55 min.

Figure 9 shows kernel density plots and box plots of the dependent variables *syntactic correctness*, *semantic correctness* and *response time* for the subset of participants with industry experience. The peak density in semantic correctness in the LTL group can be found at about $20 \%$ while the other groups have their peaks at 50–60 %. The syntactic correctness distribution of the LTL group is less steep than the ones of the other two groups with higher densities in the lower syntactic correctness ranges. While there are only minor differences in distribution shape of the response time variable between the LTL and EPL groups with their peak density in the range of 45–50 min, the PSP group has its peak density in the range of 60–65. Overall, the distribution shapes differ in central location and shape in several cases.

According to the scatter plots in Fig. 10, there is a positive linear correlation between the dependent variables *syntactic correctness* and *semantic correctness*. That is, syntactic and semantic correctness are not isolated metrics, which is not surprising, because correct application of syntax is a prerequisite for enabling meaning. There is no correlation between the correctness variables and the dependent variable *response time* (cf. Figs. 11, 12). Consequently, the amount of time spent working on the experiment tasks by the participants did not necessarily result in higher correctness values.

## Appendix C: Evaluation of normality assumption and parametric testing by Welch’s *t* test

Since the dependent variables *syntactic correctness*, *semantic correctness* and *response time* are interval-scaled, parametric methods would be the first choice, but the multivariate normality assumption appears to be violated according to the Shapiro–Wilk tests of multivariate normality in Table 22, so multivariate parametric testing (MANOVA) is ruled out. According to Shapiro–Wilk tests of univariate normality in Table 23, there are no indications of non-normality, but there are signs of non-normality in the descriptive statistics in Sect. B. Also normal QQ plots of the data show signs of non-normality (cf. Fig. 13). Due to the large sample sizes ($n > 30$) in SE2, it might be valid to assume that the Central Limit Theorem holds. In ASE, the sample size is not large enough to claim that. Since there is uncertainty regarding normality, the application of nonparametric testing should be preferred (cf. Sect. 4.3). Nonetheless, in case of assumed normality, parametric testing yields similar results (cf. Tables 24, 25). This additional testing was performed since the violation of normality is based on the interpretation of plots only, which leaves room for subjectivity.

**Table 22:** Shapiro–Wilk test of multivariate normality (* for $\alpha = 0.05$, ** for $\alpha = 0.01$, * for $\alpha = 0.001$)

Group	SE2	ASE
LTL	$W = 0.96138$	$W = 0.89909$
	$p = 0.09547$	$p = 0.03359$*
PSP	$W = 0.94299$	$W = 0.96263$
	$p = 0.02316$ *	$p = 0.6813$
EPL	$W = 0.96448$	$W = 0.91843$
	$p = 0.1618$	$p = 0.05393$

**Table 23:** Shapiro–Wilk test of univariate normality (* for $\alpha = 0.05$, ** for $\alpha = 0.01$, * for $\alpha = 0.001$)

Group	Dependent variable	SE2	ASE
LTL	Syntactic	$W = 0.97501$	$W = 0.951$
	Correctness	$p = 0.3526$	$p = 0.3558$
	Semantic	$W = 0.95487$	$W = 0.94524$
	Correctness	$p = 0.05047$	$p = 0.2761$
	Response time	$W = 0.98169$	$W = 0.95759$
		$p = 0.6127$	$p = 0.469$
PSP	Syntactic	$W = 0.96204$	$W = 0.91825$
	Correctness	$p = 0.1296$	$p = 0.138$
	Semantic	$W = 0.98311$	$W = 0.96835$
	Correctness	$p = 0.7232$	$p = 0.7889$
	Response time	$W = 0.95661$	$W = 0.89976$
		$p = 0.0789$	$p = 0.06731$
EPL	Syntactic	$W = 0.96063$	$W = 0.9358$
	Correctness	$p = 0.1139$	$p = 0.1314$
	Semantic	$W = 0.98412$	$W = 0.96757$
	Correctness	$p = 0.7652$	$p = 0.6075$
	Response time	$W = 0.98163$	$W = 0.94779$
		$p = 0.6606$	$p = 0.2425$

**Table 24:** Welch’s *t* test of syntactic/semantic correctness and response time in SE2, one-tailed with confidence intervals calculated for $\alpha = 0.05$ (cf. Welch [84]) and adjusted p-values (cf. Benjamini and Hochberg [8]) [Level of significance: * for $\alpha = 0.05$, ** for $\alpha = 0.01$, *** for $\alpha = 0.001$]

	PSP/LTL	PSP/EPL	EPL/LTL
Syntactic correctness			
*t*	3.5867	1.9529	1.5761
*df*	94.691	91.994	94.863
CI low	0.0651	0.0102	$-0.0029$
CI high	–	–	–
Mean *x*	0.6864	0.6864	0.6182
Mean *y*	0.5652	0.6182	0.5652
*p*	0.0003	0.0269	0.0592
FDR adjusted *p*	0.0013	0.0647	0.1109
Level of significance	**	–	–
Semantic correctness			
*t*	7.0831	3.8143	3.2849
*df*	93.444	91.596	95.061
CI low	0.1661	0.0679	0.048
CI high	–	–	–
Mean *x*	0.5019	0.5019	0.382
Mean *y*	0.2849	0.382	0.285
*p*	$1.3 \times 10^{-10}$	0.0001	0.0007
FDR adjusted *p*	$1.9 \times 10^{-9}$	0.0009	0.0027
Level of significance	***	***	**
Response time			
*t*	1.861	1.2971	0.5009
*df*	93.123	91.955	93.774
CI low	–	–	–
CI high	9.8170	8.6859	5.9519
*Mean* *x*	48.6769	48.6769	44.869
*Mean* *y*	43.4902	44.869	43.4902
*p*	0.9671	0.9011	0.6912
FDR adjusted *p*	0.9671	0.9655	0.7975
Level of significance	–	–	–

**Table 25:** Welch’s *t* test of syntactic/semantic correctness and response time in ASE, one-tailed with confidence intervals calculated for $\alpha = 0.05$ (cf. Welch [84]) and adjusted p-values (cf. Benjamini and Hochberg [8]) [Level of significance: * for $\alpha = 0.05$, ** for $\alpha = 0.01$, *** for $\alpha = 0.001$]

	PSP/LTL	PSP/EPL	EPL/LTL
Syntactic correctness			
*t*	1.3239	$-1.1642$	3.371
*df*	28.887	25.573	40.268
CI low	$-0.023$	$-0.1671$	0.0746
CI high	–	–	–
*Mean* *x*	0.6513	0.6513	0.7191
*Mean* *y*	0.5701	0.7191	0.5701
*p*	0.098	0.8724	0.0008
FDR adjusted *p*	0.2449	0.9254	0.0062
Level of significance	–	–	**
Semantic correctness			
*t*	3.1981	$-0.5583$	4.7839
*df*	29.231	29.156	42.581
CI low	0.0754	$-0.1125$	0.1223
CI high	–	–	–
*Mean* *x*	0.4693	0.4693	0.4971
*Mean* *y*	0.3085	0.4971	0.3085
*p*	0.0017	0.7095	$10^{-5}$
FDR adjusted *p*	0.0083	0.8869	0.0002
Level of significance	**	–	***
Response time			
*t*	0.7786	$-0.6463$	1.4701
*df*	35.654	35.389	41.049
CI lowCI low	–	–	–
CI highCI high	11.6186	4.5789	13.9503
*Mean* *x*	55.9853	55.9853	58.8236
*Mean* *y*	52.3191	58.8236	52.3191
*p*	0.7793	0.2611	0.9254
FDR adjusted *p*	0.8992	0.4352	0.9254
Level of significance	–	–	–

## Appendix D: Descriptive statistics of previous knowledge, experience and other features of participants

For the validity of the study, it is crucial to find out whether the randomized distribution to experiment groups resulted in well-balanced groups. This section provides descriptive statistics of the age, gender, programming experience, complex event processing experience, logical formalisms experience and industry experience of the participants per experiment group for that purpose.

Both the kernel density plot in Fig. 14a and the box plot in Fig. 14b show a nearly identical age distribution in all experiment groups of the SE2 course with a central tendency at 24 years. There are few (i.e., two each in LTL and PSP, and three in EPL) outliers, which represent students that are of older age than the majority of their colleagues. In contrast to the nearly identical age distribution in SE2, there seem to be minor differences in age distribution between the experiment groups of the ASE course. The kernel density plot in Fig. 14c and the box plot in Fig. 14d indicate that the share of younger participants is larger in the EPL group than in the two remaining experiment groups. Overall, LTL participants are slightly older than participants of the other groups. Moreover, there is a single age outlier in the LTL group representing a student of older age.

Figure 15 shows the gender distribution. With 111 men and 34 women, there are about three times as many male than female participants in SE2. The share of women is larger in ASE with a ratio of about 1 : 2 (21 female participants to 41 male participants). In detail, the gender distribution is as follows:

- Software Engineering 2 (SE2):
  - EPL: 12 female ($25.5 \%$) and 35 male participants ($74.5 \%$)
  - LTL: 15 female ($29.4 \%$) and 36 male participants ($70.6 \%$)
  - PSP: 7 female ($14.9 \%$) and 40 male participants ($85.1 \%$)
- Advanced Software Engineering (ASE):
  - EPL: 10 female ($41.7 \%$) and 14 male participants ($58.3 \%$)
  - LTL: 7 female ($33.3 \%$) and 14 male participants ($66.7 \%$)
  - PSP: 4 female ($23.5 \%$) and 13 male participants ($76.5 \%$)

In both courses, the share of female participants is smallest in PSP. There are about twice as many women in the LTL group in SE2 and in the EPL group in ASE as in the corresponding PSP groups, which indicates an imbalance in the distribution of female participants. Since, however, the share of women is overall low, the magnitude of potential disturbing effects is assumed to be low as well.

Figure 16 shows the participants’ programming experience. According to the kernel density plot in Fig. 16a and the box plot in Fig. 16b, the central tendency is balanced at 2-3 years in SE2. There are three outliers each in the LTL and PSP groups, and a single one in EPL, which result from participants with long-term programming experiences relatively to their colleagues in the same experiment group. In line with expectations, the participants of the master-level course ASE have more years of programming experience than their colleagues in the bachelor course SE2 (cf. Fig. 16c,d). The peak density is at 5 years programming experience in LTL and PSP. The participants of the EPL group appear to be slightly less experienced with a peak density at 4 years and a higher density in the range of 0 to 1 years. Each group has a single outlier with more years of programming experience than the other participants of the same experiment group.

Overall, the degree of industry experience is low in SE2, as to be expected in a bachelor-level course (cf. Fig. 17a, b). In ASE, a substantial amount of the students have already started to work in the industry (cf. Fig. 17c, d). Interestingly, EPL participants in ASE seem to have slightly more industry experience (cf. Fig. 17c, d) than their colleagues in the same course despite having less programming experience (cf. Fig. 16c, d).

Figure 18 shows the participants’ prior experience with Complex Event Processing (CEP). Almost all participants do not have any experience with CEP. Just two participants, one of the PSP group in SE2 and one of the EPL group in ASE, have prior experience with CEP. In contrast, the level of experience with logical formalisms is high (cf. Fig. 19). Overall, the share of experienced participants is larger in the master-level course ASE than in the bachelor-level course SE2. Interestingly, the share of prior experience with logical formalisms is higher in the LTL group than in the other groups. A potential reason could be that some of the LTL participants misunderstood this question by falsely assuming studying LTL for this experiment qualifies as “prior knowledge.”

Apart from minor differences between the experiment groups, which are to be expected in a completely randomized experiment design, the groups appear to be overall well-balanced. That is, there are no clear indications of disturbing effects on the measurement of the dependent variables resulting from unbalanced groups.

## Footnotes

1 http://nusmv.fbk.eu/.

2 http://fmt.cs.utwente.nl/tools/ltsmin/.

3 http://spinroot.com/.

4 http://www.espertech.com/esper.

5 https://github.com/wso2/siddhi.

6 http://patterns.projects.cs.ksu.edu/documentation/patterns.shtml.

7 https://www.r-project.org/.

## References

[1] van der Aalst WMP, Pesic M, Schonenberg H (2009). Declarative workflows: Balancing between flexibility and support. Comput. Sci..

[2] Awad, A., Barnawi, A., Elgammal, A., Elshawi, R., Almalaise, A., Sakr, S.: Runtime detection of business process compliance violations: An approach based on anti patterns. In: Proceedings of the 30th Annual ACM Symposium on Applied Computing. ACM, New York, SAC ’15, pp. 1203–1210 (2015) 10.1145/2695664.2699488

[3] Baier C, Katoen JP (2008). Principles of Model Checking (Representation and Mind Series).

[4] Bank for International Settlements: Basel III: International framework for liquidity risk measurement, standards and monitoring. https://www.bis.org/publ/bcbs188.htm Accessed 18 Jan 2019 (2010)

[5] Basili, V.R., Caldiera, G., Rombach, H.D.: The goal question metric approach. In: Encyclopedia of Software Engineering. Wiley (1994)

[6] Bauer A, Leucker M, Schallhart C (2010). Comparing ltl semantics for runtime verification. J. Log. Comput..

[7] Bauer A, Leucker M, Schallhart C (2011). Runtime verification for ltl and tltl. ACM Trans. Softw. Eng. Methodol..

[8] Benjamini Y, Hochberg Y (1995). Controlling the false discovery rate: a practical and powerful approach to multiple testing. J. R. Stat. Soc. B Methodol..

[9] Blom S, van de Pol J, Weber M (2010). LTSmin: Distributed and Symbolic Reachability.

[10] Carew, D., Exton, C., Buckley, J.: An empirical investigation of the comprehensibility of requirements specifications. In: 2005 International Symposium on Empirical Software Engineering (2005) 10.1109/ISESE.2005.1541834

[11] Chomsky, N. (ed.): Syntactic structures. Mouton & Co, (1957)

[12] Cimatti, .A, Clarke, E.M., Giunchiglia, E., Giunchiglia, F., Pistore, M., Roveri, M., Sebastiani, R., Tacchella, A.: Nusmv 2: An opensource tool for symbolic model checking. In: Proceedings of the 14th International Conference on Computer Aided Verification. Springer, London, CAV ’02, pp 359–364, (2002) http://dl.acm.org/citation.cfm?id=647771.734431

[13] Clarke EM, Emerson EA (1982). Design and synthesis of synchronization skeletons using branching time temporal logic.

[14] Cliff N (1993). Dominance statistics: ordinal analyses to answer ordinal questions. Psychol. Bull..

[15] Cugola, G., Margara, A.: Tesla: A formally defined event specification language. In: Proceedings of the Fourth ACM International Conference on Distributed Event-Based Systems. ACM, New York, DEBS ’10, pp 50–61 (2010) 10.1145/1827418.1827427

[16] Czepa, C., Zdun, U.: On the Understandability of Temporal Properties Formalized in Linear Temporal Logic, Property Specification Patterns and Event Processing Language [Data set]. (2017) 10.5281/zenodo.891007

[17] Czepa, C., Zdun, U.: On the understandability of temporal properties formalized in linear temporal logic, property specification patterns and event processing language. IEEE Trans. Softw. Eng. (2018) 10.1109/TSE.2018.2859926

[18] Czepa, C., Tran, H., Zdun, U., Tran, T., Weiss, E., Ruhsam, C.: Plausibility checking of formal business process specifications in linear temporal logic. In: 28th International Conference on Advanced Information Systems Engineering (CAiSE’16), Forum Track, http://eprints.cs.univie.ac.at/4692/

[19] Czepa, C., Tran, H., Zdun, U., Tran, T., Weiss, E., Ruhsam, C.: Plausibility checking of formal business process specifications in linear temporal logic (extended abstract). In: Mendling, J., Rinderle-Ma, S. (eds) 7th International Workshop on Enterprise Modeling and Information Systems Architectures (EMISA 2016), (2016b) http://eprints.cs.univie.ac.at/4780/

[20] Czepa, C., Tran, H., Zdun, U., Tran, T., Weiss, E., Ruhsam, C.: Towards a compliance support framework for adaptive case management. In: 5th International Workshop on Adaptive Case Management and other Non-workflow Approaches to BPM (AdaptiveCM 16), 20th IEEE International Enterprise Computing Workshops (EDOCW 2016), (2016c) http://eprints.cs.univie.ac.at/4752/

[21] Czepa, C., Tran, H., Zdun, U., Tran, T., Weiss, E., Ruhsam, C.: On the understandability of semantic constraints for behavioral software architecture compliance: A controlled experiment. In: IEEE International Conference on Software Architecture (ICSA 2017), (2017) http://eprints.cs.univie.ac.at/5059/

[22] Czepa, C., Amiri, A., Ntentos, E., Zdun, U.: Modeling Compliance Specifications in Linear Temporal Logic, Event Processing Language and Property Specification Patterns [Data set]. (2018) 10.5281/zenodo.1246561 10.1007/s10270-019-00721-4 PMC6944266 31975976

[23] De Giacomo, G., Vardi, M.Y.: Linear temporal logic and linear dynamic logic on finite traces. In: Proceedings of the Twenty-Third International Joint Conference on Artificial Intelligence. AAAI Press, IJCAI ’13, pp. 854–860, (2013) http://dl.acm.org/citation.cfm?id=2540128.2540252

[24] De Giacomo G, De Masellis R, Grasso M, Maggi FM, Montali M, Sadiq S, Soffer P, Völzer H (2014). Monitoring business metaconstraints based on ltl and ldl for finite traces. Business Process Management.

[25] De Giacomo, G., De Masellis, R., Montali, M.: Reasoning on ltl on finite traces: Insensitivity to infiniteness. In: Proceedings of the Twenty-Eighth AAAI Conference on Artificial Intelligence. AAAI Press, AAAI’14, pp. 1027–1033, (2014b) http://dl.acm.org/citation.cfm?id=2893873.2894033

[26] De Smedt J, De Weerdt J, Serral E, Vanthienen J (2016). Improving Understandability of Declarative Process Models by Revealing Hidden Dependencies.

[27] Dwyer, M.B., Avrunin, G.S., Corbett, J.C.: Property specification patterns for finite-state verification. In: Proceedings of the Second Workshop on Formal Methods in Software Practice, ACM, New York, FMSP ’98, pp. 7–15, (1998) 10.1145/298595.298598

[28] Dwyer, M.B., Avrunin, G.S., Corbett, J.C.: Patterns in property specifications for finite-state verification. In: Proceedings of the 21st International Conference on Software Engineering. ACM, New York, ICSE ’99, pp 411–420, (1999) 10.1145/302405.302672

[29] Elgammal A, Turetken O, van den Heuvel WJ, Papazoglou M (2016). Formalizing and appling compliance patterns for business process compliance. Softw. Syst. Model..

[30] EsperTech Inc: EPL Reference. http://www.espertech.com/esper/release-6.0.1/esper-reference/html/event_patterns.html. Accessed 18 Jan 2019 (2017)

[31] Feigenspan J, Kästner C, Apel S, Liebig J, Schulze M, Dachselt R, Papendieck M, Leich T, Saake G (2013). Do background colors improve program comprehension in the #ifdef hell?. Empir. Softw. Eng..

[32] Ferri, F., Pourabbas, E., Rafanelli, M.: The syntactic and semantic correctness of pictorial configurations to query geographic databases by pql. In: Proceedings of the 2002 ACM Symposium on Applied Computing. ACM, New York, SAC ’02, pp 432–437, (2002) 10.1145/508791.508873

[33] Fox, J., Weisberg, S.: An R Companion to Applied Regression, 2nd edn. Sage, Thousand Oaks CA (2011), http://socserv.socsci.mcmaster.ca/jfox/Books/Companion

[34] Habiballa H, Kmet T (2004). Theoretical branches in teaching computer science. Int. J. Math. Educ. Sci. Technol..

[35] Haisjackl C, Zugal S (2014). Investigating Differences between Graphical and Textual Declarative Process Models.

[36] Haisjackl C, Zugal S, Soffer P, Hadar I, Reichert M, Pinggera J, Weber B (2013). Making Sense of Declarative Process Models: Common Strategies and Typical Pitfalls.

[37] Harel, D., Rumpe, B.: Modeling languages: Syntax, semantics and all that stuff, part i: The basic stuff. Tech. rep, Jerusalem, Israel, Israel (2000)

[38] Heijstek, W., Kuhne, T., Chaudron, M.R.V.: Experimental analysis of textual and graphical representations for software architecture design. In: 2011 International Symposium on Empirical Software Engineering and Measurement, pp. 167–176, (2011) 10.1109/ESEM.2011.25

[39] Hindawi M, Morel L, Aubry R, Sourrouille JL, Chaudron MRV (2009). Description and implementation of a uml style guide. Models in Software Engineering.

[40] Hoisl, B., Sobernig, S., Strembeck, M.: Comparing three notations for defining scenario-based model tests: A controlled experiment. In: QUATIC’14, pp 95–104, (2014)

[41] Holmes T, Mulo E, Zdun U, Dustdar S (2011). Model-aware Monitoring of SOAs for Compliance.

[42] Holzmann GJ (1997). The model checker spin. IEEE Trans. Softw. Eng..

[43] Höst M, Regnell B, Wohlin C (2000). Using students as subjects–a comparative study of students and professionals in lead-time impact assessment. Empir. Softw. Eng..

[44] Bryer, Jason, Speerschneider, Kimberly: likert: Analysis and Visualization Likert Items. https://CRAN.R-project.org/package=likert. Accessed 18 Jan 2019 (2016)

[45] Jedlitschka A, Ciolkowski M, Pfahl D (2008). Reporting Experiments in Software Engineering.

[46] Jot, G., Huber, J., Heriko, M., Polani, G.: An empirical investigation of intuitive understandability of process diagrams. Comput. Stand. Interfaces **48**, 90–111 (2016). 10.1016/j.csi.2016.04.006, http://www.sciencedirect.com/science/article/pii/S0920548916300332, special Issue on Information System in Distributed Environment

[47] Juristo N, Moreno AM (2010). Basics of Software Engineering Experimentation.

[48] Khoshafian, S.: Intelligent BPM: The Next Wave for Customer Centric Business Applications. Pegasystems Incorporated, (2013) https://books.google.at/books?id=IYACnwEACAAJ

[49] Kitchenham B, Madeyski L, Budgen D, Keung J, Brereton P, Charters S, Gibbs S, Pohthong A (2016). Robust statistical methods for empirical software engineering. Empir. Softw. Eng..

[50] Kitchenham BA, Pfleeger SL, Pickard LM, Jones PW, Hoaglin DC, Emam KE, Rosenberg J (2002). Preliminary guidelines for empirical research in software engineering. IEEE Trans. Softw. Eng..

[51] Knobelsdorf, M., Frede, C.: Analyzing student practices in theory of computation in light of distributed cognition theory. In: Proceedings of the 2016 ACM Conference on International Computing Education Research. ACM, New York, ICER ’16, pp. 73–81, (2016) 10.1145/2960310.2960331

[52] Kowalski R, Sergot M (1986). A logic-based calculus of events. New Gener. Comput..

[53] Lytra, I., Gaubatz, P., Zdun, U.: Two controlled experiments on model-based architectural decision making. Inf. Softw. Technol. **58**, 63–75 (2015). 10.1016/j.infsof.2015.03.006, http://eprints.cs.univie.ac.at/4342/

[54] Maggi FM, Westergaard M, Montali M, van der Aalst WMP, Khurshid S, Sen K (2012). Runtime verification of ltl-based declarative process models. Runtime Verification.

[55] Michael, G Oxley: H.R.3763 - Sarbanes-Oxley Act of 2002. https://www.congress.gov/bill/107th-congress/house-bill/3763/text. Accessed 18 Jan 2019 (2002)

[56] Montali M (2010). The ConDec Language.

[57] Montali M, Maggi FM, Chesani F, Mello P, Aalst WMPvd (2014). Monitoring business constraints with the event calculus. ACM Trans. Intell. Syst. Technol..

[58] Naimi, B., as Hamm, N., Groen, T.A., Skidmore, A.K., Toxopeus, AG.: Where is positional uncertainty a problem for species distribution modelling. Ecography **37**, 191–203 (2014). 10.1111/j.1600-0587.2013.00205.x

[59] OMG: BPMN 2.0. http://www.omg.org/spec/BPMN/2.0/PDF. Accessed 18 Jan 2019, (2011)

[60] OMG: Semantics of Business Vocabulary and Rules (SBVR). http://www.omg.org/spec/SBVR/. Accessed 18 Jan 2019 (2017)

[61] Pesic, M., Schonenberg, H., van der Aalst, W.M.P.: Declare: Full support for loosely-structured processes. In: Proceedings of the 11th IEEE International Enterprise Distributed Object Computing Conference, IEEE Computer Society, Washington, EDOC ’07, (2007) http://dl.acm.org/citation.cfm?id=1317532.1318056

[62] Pešić M, Bošnački D, van der Aalst WMP, van de Pol J, Weber M (2010). Enacting declarative languages using ltl: Avoiding errors and improving performance. Model Checking Software.

[63] Filzmoser, Peter, Gschwandtner, Moritz: mvoutlier: Multivariate Outlier Detection Based on Robust Methods. https://CRAN.R-project.org/package=mvoutlier. Accessed 18 Jan 2019, (2017)

[64] Pichler P, Weber B, Zugal S, Pinggera J, Mendling J, Reijers HA (2012). Imperative versus Declarative Process Modeling Languages: An Empirical Investigation.

[65] Pnueli, A.: The temporal logic of programs. In: Proceedings of the 18th Annual Symposium on Foundations of Computer Science, IEEE Computer Society, Washington, SFCS ’77, pp. 46–57, (1977) 10.1109/SFCS.1977.32

[66] Reichert M, Weber B (2012). Enabling Flexibility in Process-Aware Information Systems: Challenges, Methods, Technologies.

[67] Revelle, W.: psych: Procedures for Psychological, Psychometric, and Personality Research. Northwestern University, Evanston, Illinois, https://CRAN.R-project.org/package=psych, r package version 1.7.5, (2017)

[68] Revilla MA, Saris WE, Krosnick JA (2014). Choosing the number of categories in agreedisagree scales. Sociol. Methods Res..

[69] Rodrigues, R.D., Barros, M.D., Revoredo, K., Azevedo, L.G., Leopold, H.: An experiment on process model understandability using textual work instructions and bpmn models. In: 2015 29th Brazilian Symposium on Software Engineering (SBES), vol 00, pp. 41–50, (2015) 10.1109/SBES.2015.12

[70] Rogmann, J.J.: Ordinal dominance statistics (orddom): An r project for statistical computing package to compute ordinal, nonparametric alternatives to mean comparison (version 3.1). Available online from the CRAN website, (2013) http://cran.r-project.org/

[71] Rovani, M., Maggi, F.M., de Leoni, M., van der Aalst, W.M.: Declarative process mining in healthcare. Exp. Syst. Appl. **42**(23), 9236–9251 (2015). 10.1016/j.eswa.2015.07.040, http://www.sciencedirect.com/science/article/pii/S095741741500500X

[72] Runeson, P.: Using students as experiment subjects an analysis on graduate and freshmen student data. In: Proceedings 7th International Conference on Empirical Assessment and Evaluation in Software Engineering, pp. 95–102, (2003)

[73] Salman, I., Misirli, A.T., Juristo, N.: Are students representatives of professionals in software engineering experiments? In: Proceedings of the 37th International Conference on Software Engineering - Volume 1, IEEE Press, Piscataway, ICSE ’15, pp. 666–676, (2015) http://dl.acm.org/citation.cfm?id=2818754.2818836

[74] Sharafi, Z., Marchetto, A., Susi, A., Antoniol, G., Guhneuc, Y.G.: An empirical study on the efficiency of graphical versus textual representations in requirements comprehension. In: 2013 21st International Conference on Program Comprehension (ICPC), pp. 33–42, (2013) 10.1109/ICPC.2013.6613831

[75] da Silva, A.R., Malafaia, G., de Menezes, I.P.P.: biotools: an r function to predict spatial gene diversity via an individual-based approach. Genet. Mol. Res. 16:gmr16029,655, (2017) 10.4238/gmr16029655 28407196

[76] Jarek, Slawomir: mvnormtest: Normality test for multivariate variables. https://CRAN.R-project.org/package=mvnormtest. Accessed 18 Jan 2019 (2012)

[77] Spichkova M, Milazzo P, Varró D, Wimmer M (2016). “boring formal methods” or “sherlock holmes deduction methods”?. Software Technologies: Applications and Foundations.

[78] Svahnberg, M., Aurum, A., Wohlin, C.: Using students as subjects - an empirical evaluation. In: Proceedings of the Second ACM-IEEE International Symposium on Empirical Software Engineering and Measurement, ACM, New York, ESEM ’08, pp. 288–290, (2008) 10.1145/1414004.1414055

[79] Tran, T., Weiss, E., Ruhsam, C., Czepa, C., Tran, H., Zdun, U.: Embracing process compliance and flexibility through behavioral consistency checking in ACM: A repair service management case. In: 4th International Workshop on Adaptive Case Management and other Non-workflow Approaches to BPM (AdaptiveCM 15), Business Process Management Workshops 2015, (2015a) http://eprints.cs.univie.ac.at/4409/

[80] Tran, T., Weiss, E., Ruhsam, C., Czepa, C., Tran, H., Zdun, U.: Enabling flexibility of business processes by compliance rules: A case study from the insurance industry. In: 13th International Conference on Business Process Management 2015, Industry Track, (2015b) http://eprints.cs.univie.ac.at/4399/

[81] Tran, T., Weiss, E., Adensamer, A., Ruhsam, C., Czepa, C., Tran, H., Zdun, U.: An ontology-based approach for defining compliance rules by knowledge workers in adaptive case management. In: 5th International Workshop on Adaptive Case Management and other Non-workflow Approaches to BPM (AdaptiveCM 16), 20th IEEE International Enterprise Computing Workshops (EDOCW 2016), (2016) http://eprints.cs.univie.ac.at/4753/

[82] Tran, T., Weiss, E., Ruhsam, C., Czepa, C., Tran, H., Zdun, U.: Enabling flexibility of business processes using compliance rules: The case of Mobiliar. In: vom Brocke, J., Mendling, J. (eds.) Business Process Management Cases. Springer, Berlin (2017). http://eprints.cs.univie.ac.at/5094/

[83] United States Environmental Protection Agency: EPAs Lead-Based Paint Renovation, Repair and Painting (RRP) Rule. https://www.epa.gov/lead/lead-renovation-repair-and-painting-program-rules. Accessed 18 Jan 2019 (2011)

[84] Welch BL (1947). The generalization of student’s problem when several different population variances are involved. Biometrika.

[85] Wickham, H.: ggplot2: Elegant Graphics for Data Analysis. Springer, New York (2009), http://ggplot2.org

[86] Wohlin C, Runeson P, Höst M, Ohlsson MC, Regnell B, Wesslén A (2000). Experimentation in Software Engineering: An Introduction.

[87] Zugal S, Soffer P, Haisjackl C, Pinggera J, Reichert M, Weber B (2015). Investigating expressiveness and understandability of hierarchy in declarative business process models. Softw. Syst. Model..

